# Identifying the essential nutritional requirements of the probiotic bacteria *Bifidobacterium animalis* and *Bifidobacterium longum* through genome-scale modeling

**DOI:** 10.1038/s41540-021-00207-4

**Published:** 2021-12-09

**Authors:** Marie Schöpping, Paula Gaspar, Ana Rute Neves, Carl Johan Franzén, Ahmad A. Zeidan

**Affiliations:** 1 Systems Biology, Discovery, Chr. Hansen A/S, 2970 Hørsholm, Denmark; 2 Division of Industrial Biotechnology, Department of Biology and Biological Engineering, Chalmers University of Technology, 41296 Gothenburg, Sweden; 3 Present Address: Arla Foods Ingredients Group P/S, 6920 Videbæk, Denmark

**Keywords:** Biochemical networks, Physiology

## Abstract

Although bifidobacteria are widely used as probiotics, their metabolism and physiology remain to be explored in depth. In this work, strain-specific genome-scale metabolic models were developed for two industrially and clinically relevant bifidobacteria, *Bifidobacterium animalis* subsp. *lactis* BB-12^®^ and *B. longum* subsp. *longum* BB-46, and subjected to iterative cycles of manual curation and experimental validation. A constraint-based modeling framework was used to probe the metabolic landscape of the strains and identify their essential nutritional requirements. Both strains showed an absolute requirement for pantethine as a precursor for coenzyme A biosynthesis. Menaquinone-4 was found to be essential only for BB-46 growth, whereas nicotinic acid was only required by BB-12^®^. The model-generated insights were used to formulate a chemically defined medium that supports the growth of both strains to the same extent as a complex culture medium. Carbohydrate utilization profiles predicted by the models were experimentally validated. Furthermore, model predictions were quantitatively validated in the newly formulated medium in lab-scale batch fermentations. The models and the formulated medium represent valuable tools to further explore the metabolism and physiology of the two species, investigate the mechanisms underlying their health-promoting effects and guide the optimization of their industrial production processes.

## Introduction

Bifidobacteria are Gram-positive, obligate anaerobic prokaryotes that commonly inhabit the gastrointestinal tract of humans and animals. The presence of bifidobacteria is associated with a healthy gastrointestinal tract and a strong immune function of the host. Health-promoting effects of bifidobacteria include the production of antimicrobial agents and vitamins^1^, the inhibition of adhesion of pathogens and toxins to epithelial cells, and the stimulation of the host’s immune response^2,3^. Based on their beneficial impact on health, several *Bifidobacterium* strains, such as *Bifidobacterium animalis* subsp. *lactis* BB-12^®^, are commercially applied as probiotics in food and pharmaceutical products^3,4^.

**Fig. 1:**
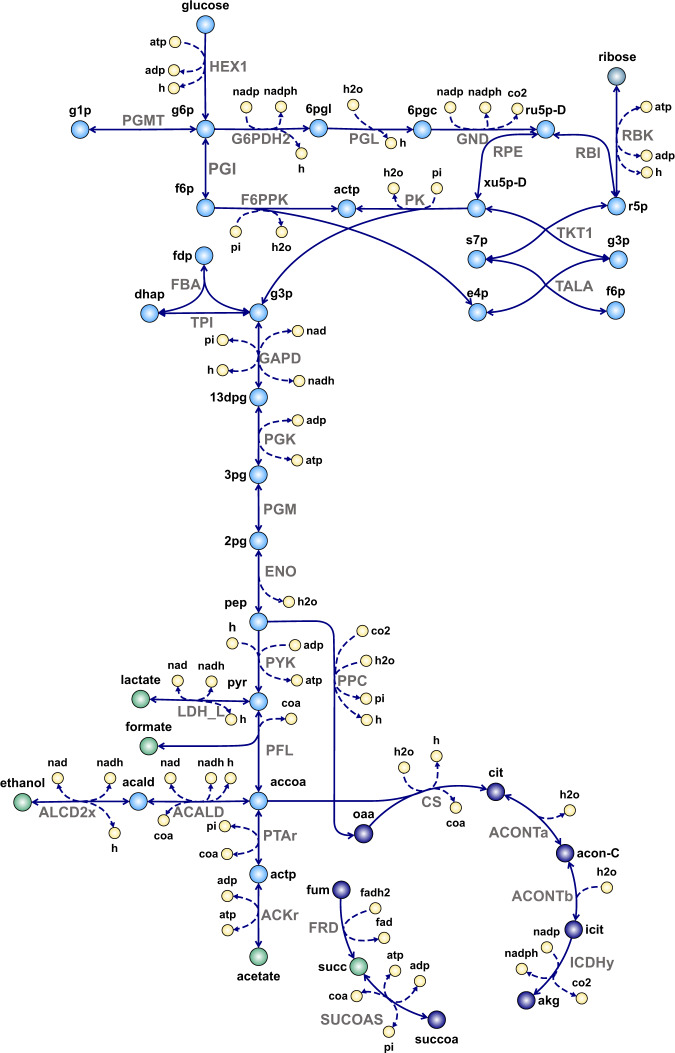
Central carbon metabolism in bifidobacteria, as depicted in *i*AZ480 and *i*MS520. Abbreviations of metabolite and reaction names are the same as used in the models (Supplementary Data 3 and 4).

The physiological characteristics and metabolic capabilities can vary significantly among *Bifidobacterium* species and strains^5,6^. However, all bifidobacteria use a characteristic hexose fermentation pathway, known as the bifid shunt, for carbon dissimilation^4,7^, due to the lack of 6-phosphofructokinase (EC 2.7.1.11), a key enzyme of the canonical Embden-Meyerhof-Parnas pathway. This heterofermentative pathway relies on the key enzyme fructose-6-phosphate phosphoketolase (Xfp)^7^. Xfp has a dual substrate specificity^8^ and can catalyze the conversion of both fructose-6-phosphate and xylulose-5-phosphate to acetyl phosphate and the corresponding aldose phosphate (F6PPK, EC 4.1.2.22 and PK, EC 4.1.2.9), which are further catabolized towards different fermentation products (Fig. 1). Theoretically, the fermentation of two molecules of glucose yields three moles of acetate and two moles of lactate. However, these theoretical yields are rarely observed experimentally^9–17^. Besides acetate and lactate, formate, ethanol, and succinate can also be secreted from bifidobacteria^9–13,15,17^. The profile of end product formation seems to depend on the carbon source^9–11,14,17^, carbon availability^13,15^, the specific consumption rate of the carbon source^12^, as well as on the *Bifidobacterium* strain^9,12,13^. Changes in the flux split ratios impact the final ATP yield on consumed substrate as well as the NADH balance; additional lactate and ethanol formation contribute to regeneration of NADH that is produced in the oxidative part of the glycolysis, while displacement of the bifid shunt towards acetate production is linked to the formation of an extra ATP^15,17,18^ (Fig. 1). An increase in ethanol formation at the expense of lactate increases ATP production^10^ due to the regeneration of an additional NADH that frees carbon for ATP formation via acetate formation (Fig. 1). In general, the bifid shunt allows the production of more ATP per hexose than other glycolytic pathways do^10^.

The central carbon metabolism of bifidobacteria has been investigated by quantitative analysis of fermentation end products^9–15^, determination of metabolic flux distribution using ^13^C-nuclear magnetic resonance (NMR) spectroscopy^16,17^, and examination of catalytic activities of individual enzymes^8,15,17^. However, systems-level metabolic and physiological characteristics of bifidobacteria remain yet to be explored in greater depth. These include the essential nutritional requirements of bifidobacteria and the underlying genetic basis of their biosynthetic capabilities. As bifidobacteria are rather fastidious microorganisms, most previous studies used either complex undefined media^10,12,19,20^ or semi-defined media supplemented with complex additives, such as (hydrolyzed) casein or the permeate of dialyzed complex medium^21,22^, to sustain growth. While such media allow for high growth rates and high final biomass concentrations, they are not suitable for detailed physiological studies since complex medium components with undefined composition may hide the effect of a metabolite and hamper the quantification of product yields and growth energetics.

Currently, industrial production processes of probiotics, including bifidobacteria, are typically optimized based on empirical design approaches. While it is desirable to adopt more rational, knowledge-driven approaches for identifying the essential nutritional requirements and advancing industrial-scale production, necessary tools such as genome-scale metabolic models (GEMs) are yet to be developed. The reconstruction of high-quality strain-specific genome-scale metabolic networks is a powerful approach in microbial systems biology, since it leverages the value of the exponentially growing genomics data, enabling its integration with other biological knowledge in a structured format^23^. Converting a network reconstruction into a GEM enables the quantitative and qualitative analysis of the reconstructed metabolic network using constraint-based methods. Although GEMs for some *Bifidobacterium* strains have been previously developed, their value is confined by their quality and scope, as they have been subjected to a limited amount of manual curation and/or experimental validation^24–26^.

In this work, we constructed and manually curated strain-specific GEMs for two industrially important probiotic strains, *B. animalis* subsp. *lactis* BB-12^®^ (hereafter abbreviated BB-12) and *B. longum* subsp. *longum* BB-46 (hereafter abbreviated BB-46). The GEMs are capable of accurately describing and predicting the metabolic capabilities of these strains. We present the general features of these manually curated GEMs and describe how they were employed to probe the metabolic landscape and identify the individual nutritional requirements of the two strains. Model-generated insights were used to guide the formulation of a chemically defined medium (CDM) that supports the growth of both strains in the same manner as complex medium. Finally, the models were quantitatively validated in the newly developed medium in lab-scale batch fermentations.

## Results and Discussion

### Genome sequence of *B. longum* BB-46

The hybrid assembly of *B. longum* BB-46 genome resulted in a single 2.385.558-bp circular chromosome with an average G + C content of 60.33%. A total of 1870 predicted protein-encoding sequences, 77 tRNA genes, and 4 rRNA operons were identified. No plasmids could be detected in the strain. The complete genome sequence was deposited in NCBI GenBank (Accession no. CP065209). Sequencing statistics and other relevant characteristics of the genome sequence are summarized in Supplementary Table 1.

### Characteristics of BB-12 and BB-46 genome-scale metabolic models

**Fig. 2:**
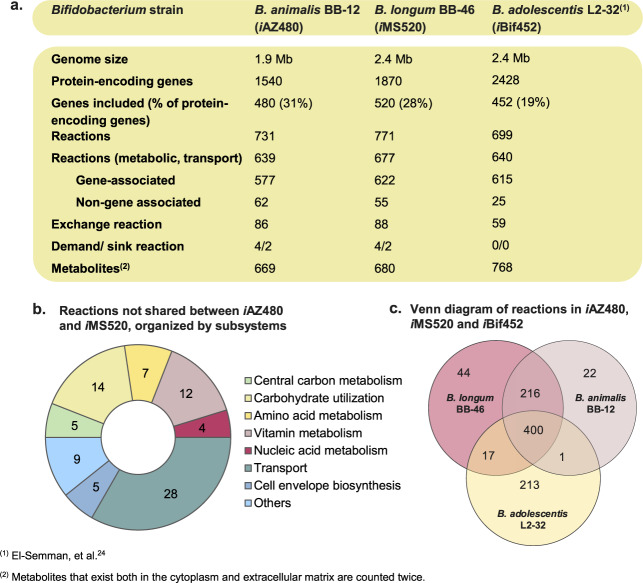
Comparison of *i*AZ480, *i*MS520, and *i*Bif452. **a** Main characteristics of the metabolic networks of *i*AZ480 and *i*MS520 and *i*Bif452. **b** Overview of unique reactions in *i*AZ480 and *i*MS520, which exist only in one of the models but not in the other. **c** A Venn diagram representing the reactions included in *i*AZ480, *i*MS520, and *i*Bif452. The comparison was performed based on BiGG reaction identifiers and EC numbers.

The refined GEM of *B. animalis* BB-12 (hereafter referred to as *i*AZ480) contained 731 reactions and 480 genes, whereas that of *B. longum* BB-46 (hereafter referred to as *i*MS520) contained 771 reactions and 520 genes (Fig. 2a). In agreement with the smaller genome size of BB-12, *i*AZ480 includes 5% fewer reactions and 8% fewer genes than *i*MS520. Sixty-two reactions in *i*AZ480 and 55 reactions in *i*MS520 are not associated with any genes; around half of these reactions are transport reactions that lack a gene association due to missing knowledge on transporter specificity. A total of 84 reactions are not shared between *i*AZ480 and *i*MS520, mainly including transport reactions, vitamin and amino acid biosynthesis, and carbohydrate utilization (Fig. 2b). Both GEMs reached a MEMOTE^27^ score of 83% (Supplementary Data 1 and 2). The complete lists of metabolic reactions, metabolites and associated genes included in *i*AZ480 and *i*MS520 are available in Excel format in Supplementary Data 3 and 4, as well as in Systems Biology Markup Language (SBML) format in Supplementary Data 5 and 6.

In comparison to *i*AZ480 and *i*MS520, the previously published GEM of *Bifidobacterium adolescentis* L2-32 (*i*Bif452)^24^ covers a lower percentage of the protein-encoding genes in the strain’s genome and includes a significantly smaller number of non-gene assigned reactions (Fig. 2a). The three GEMs were compared based on BiGG reaction identifiers and EC numbers, with exchange reactions excluded. While *i*AZ480 and *i*MS520 share the majority of reactions with *i*Bif452, the latter contains a significant number of unique reactions (Fig. 2c), most of which are associated with membrane transport and the biosynthesis of cell envelope components. The transport mechanism of a substrate might partly differ in the GEMs due to limited accuracy of transporter annotation. Alternatively, this could be seen as an indication of a wide variation of transporters in bifidobacteria. In contrast to *i*AZ480 and *i*MS520, *i*Bif452 includes a simplified, non-genus-specific biomass objective function (BOF). Some variations in the reconstruction of the pathways for central carbon and amino acid metabolism in *i*Bif452 from *i*AZ480 and *i*MS520 are discussed in later sections.

### Draft reconstruction and manual curation

The draft network reconstruction of BB-12 generated by Model SEED originally included 727 reactions, in addition to a generic biomass formation reaction. During the refinement of the reconstruction, a total of 287 reactions were added to the network based on biochemical, physiological, or genomic evidence, whereas 339 reactions were removed, as their presence in the strain could not be verified. Many of the removed reactions where non-specific reactions that are not known to occur in anaerobic bacteria in general or bifidobacteria in particular. Other reactions were removed, since their associated genes were assigned to different reactions during manual curation. This clearly highlights the importance of curating automated network reconstructions for incorporating organism-specific knowledge and eliminating unvalidated generic reactions.

To create a network reconstruction for BB-46, 526 gene-associated and non-gene-associated reactions and 79 exchange reactions were carried over from the metabolic network of BB-12. Based on various evidence, new reactions were added to BB-46 reconstruction, genes could be assigned to non-gene associated reactions and the gene-protein-reaction (GPR) associations were generally updated. A complete overview of reactions that were added, removed, or changed in the draft reconstruction of BB-12 and BB-46 is provided in Supplementary Data 7.

#### Central carbon metabolism

Reactions of the bifid shunt, the pentose-phosphate pathway (PPP) and the tricarboxylic acid cycle (TCA) cycle included in the refined reconstructions of both strains (Fig. 1) were inferred from genomics data as well as physiological data^4,8,12,17,28^.

Based on a previous ^13^C-NMR pathway analysis in BB-12^17^, the pyruvate formate-lyase (EC 2.3.1.54) reaction (PFL) was made reversible, allowing both the formation of acetyl-CoA and its condensation with formate to generate pyruvate. The alternative route of generating acetyl-CoA via pyruvate dehydrogenase was not included in the GEMs, since both strains lack the genes encoding the complete pyruvate dehydrogenase enzyme complex.

BB-12 and BB-46 possess an incomplete TCA cycle, as both strains lack genes for malate dehydrogenase (EC 1.1.1.37), fumarase (EC 4.2.1.2), and the 2-oxoglutarate dehydrogenase enzyme complex. Examining the presence of TCA cycle enzymes in other bifidobacteria in the KEGG database^29^ showed that an incomplete TCA cycle is a common feature in these bacteria. All sequenced bifidobacteria seem to lack the same set of genes, except for *Bifidobacterium dentium* Bd1, which possesses a gene for malate dehydrogenase. In contrast to *i*AZ480 and *i*MS520, *i*Bif452 includes pyruvate dehydrogenase and 2-oxoglutarate dehydrogenase reactions, which are both assigned to the dihydrolipoamide dehydrogenase gene^24^. This highlights an inherent issue in automated network reconstructions based on KEGG pathways, since the KEGG database considers the presence of the genes for any of the enzymes in a multienzyme complex as an indication of the presence of the corresponding reaction.

#### Biomass objective function

A *Bifidobacterium*-specific BOF was formulated based on the macromolecular composition of BB-12 determined in this study, as well as data from literature on bifidobacteria and closely related species. The protein content of BB-12 (52.2 ± 0.6%; w/w) as well as its DNA content (3.77 ± 1.0%, w/w) is higher than the corresponding fractions in other Gram-positive bacteria, e.g., *Lactococcus lactis*^30^, *Lactobacillus plantarum*^31^, and *Streptococcus thermophilus*^32^, while its RNA content (4.9 ± 0.5%; w/w) is comparatively low. However, the deviations might be explained by the use of different growth conditions, which are known to affect the macromolecular composition of bacterial cells^33^. For BB-46, the peptidoglycan composition in the BOF was adjusted to reflect its cell wall type. The resulting BOF was incorporated into the metabolic networks of BB-12 and BB-46. Based on the BOF, the elemental composition of the cells is CH_1.57_N_0.23_O_0.43_P_0.01_, corresponding to a molecular weight of 24.7 g C-mol^−1^. Further detailed information on the cellular composition and the formulation of the BOF is provided in Supplementary Note 1.

#### In silico network evaluation

The extensive refinement of the reconstructed networks in a pathway-by-pathway manner resulted in immediately functional GEMs that could predict non-zero flux through the biomass reaction when performing flux balance analysis (FBA)^34^. None of the GEMs produced matter or energy from nothing when optimizing for the BOF while constraining all exchange rates. FBA revealed the presence of a number of internal loops (type III extreme cycles^35^) among the reactions, listed in Supplementary Note 2. To enhance the completeness of the GEMs, we identified metabolic dead ends in the networks and searched for genomic or experimental evidence to fill missing metabolic functions. Examples of gap-filling reactions are listed in Supplementary Note 3.

#### Verification of known metabolic functions

*i*AZ480 and *i*MS520, were further evaluated by comparing FBA predictions to known metabolic characteristics of bifidobacteria. Sucrose was used as the carbon source in the simulations with its uptake rate constrained to 10 mmol g_CDW_^−1^h^−1^, where CDW is the cell dry weight, whereas the uptake rates of all amino acids, vitamins, and nucleobases were unconstrained. When maximizing for biomass formation, the GEMs predicted unlikely high uptake rates of some amino acids, resulting in unrealistically high growth rates. The use of amino acids as additional carbon source in *i*AZ480 and *i*MS520 could be attributed to the activity of serine deaminase (EC 4.3.1.17) or serine-specific threonine-ammonia lyase (EC 4.3.1.19) as well as alanine transaminases (EC 2.6.1.2), converting the corresponding amino acids into intermediates in the central carbon metabolism and further to acetate for ATP synthesis. When the sucrose uptake was constrained to zero, no growth on amino acids as the sole carbon and energy source was predicted, which is in agreement with previous knowledge on bifidobacteria^36^. When constraining the uptake rates of all amino acids to 1 mmol g_CDW_^−1^ h^−1^, *i*AZ480 and *i*MS520 predicted the formation of 1.8 mol acetate, 1.0 mol formate, 0.4 mol ethanol, and 3 mol ATP per mol hexose consumed, but no lactate secretion was observed. The absence of lactate formation and a higher acetate:lactate ratio than the theoretical ratio of 3:2 have been previously observed in bifidobacteria^9–13,15,17^, indicating that acetate formation is preferred under certain conditions, since it is linked to additional ATP generation^18^. When the acetate:lactate ratio was constrained to the theoretical ratio of 3:2, both models predicted the secretion of 0.9 mol lactate, 1.3 mol acetate, and minor amounts of formate per mol of hexose, while the ATP yield slightly dropped to 2.9 mol per mol hexose. A similar end-product profile was obtained by constraining the secretion of formate to zero, which restricts the flux through PFL. With the same constraint on formate secretion for growth on pentoses, *i*AZ480 and *i*MS520 predicted an acetate:lactate ratio of 1:1. The lower acetate production is attributed to pentoses entering the bifid shunt directly via xylulose-5-phosphate, eliminating the possibility of additional acetyl–phosphate formation through the fructose-6-phosphate phosphoketolase (F6PPK/PK) reaction (Fig. 1)^37^. Under all tested conditions, slightly higher growth rates were predicted for BB-46 than for BB-12 (2–7%).

Both *i*AZ480 and *i*MS520 predicted the secretion of small amounts of succinate, which has been previously observed in bifidobacteria and was suggested to occur from phosphoenolpyruvate via the reduction of oxaloacetate, malate and fumarate^12^. Due to the absence of malate dehydrogenase and fumarase, *i*AZ480 and *i*MS520 predicted succinate production from fumarate either through fumarate reductase (EC 1.3.5.4) in the TCA cycle or via fumarate-dependent dihydroorotic acid dehydrogenase (EC 1.3.98.1) in pyrimidine biosynthesis. According to model predictions, the required fumarate may be formed in the adenylosuccinate lyase (EC 4.3.2.2) reaction in the purine metabolism. Apart from being secreted, succinate also serves as the direct precursor for succinyl-CoA formation by succinyl-CoA synthetase (EC 6.2.1.5) (SUCOAS reaction, Fig. 1).

### Experimental validation and iterative refinement

**Fig. 3:**
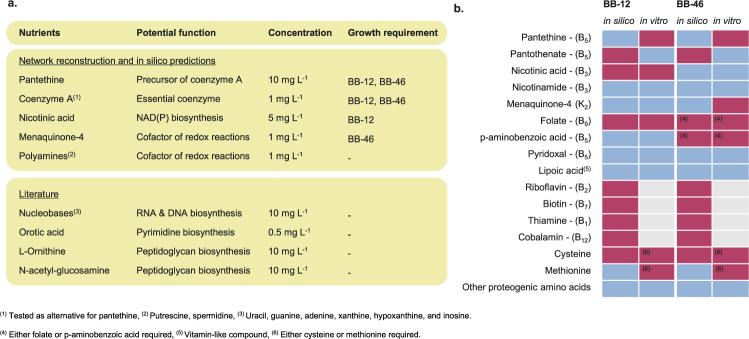
Investigation of the essential nutritional requirements of BB-12 and BB-46. **a** Potentially essential nutrients for growth of BB-12 and BB-46 that are missing in the generic culture medium, as identified through in silico analysis and literature. The requirement for different nutrients was evaluated in vitro through nutrient omission experiments. **b** Heat map comparing the in silico predictions and the in vitro results regarding vitamin and amino acid requirements of BB-12 and BB-46. Blue: not essential, red: essential, gray: not tested. Knowledge gained from the validation experiments was incorporated into *i*AZ480 and *i*MS520 through the addition of new reactions or deletion of existing reactions so that the model predictions agree with the in vitro results.

For the experimental validation of *i*AZ480 and *i*MS520, we aimed at conducting growth experiments in a CDM to allow the quantitative characterization of growth and metabolite production and enable the investigation of the influence of specific nutrients. However, growth of BB-12 and BB-46 could not be supported on CDMs previously reported for bifidobacteria^38–41^. A generic CDM including all basic microbial growth requirement could not sustain the growth of BB-12 and BB-46 over multiple subcultures either. Therefore, we used the models to predict the nutritional requirements of each strain through in silico single omission experiments. In addition, we tested how the removal of gap-filling reactions affected the predictions and consulted available data on nutritional requirements of bifidobacteria from literature^38,42,43^. Following this approach, 15 nutrients that were missing in the generic CDM and could potentially be essential for growth of one or both strains were identified (Fig. 3a). When supplemented with the 15 nutrients, the CDM sustained reproducible growth of BB-12 and BB-46 over three passages, proving that the enriched medium provides all essential nutrients for growth of the strains. Both strains reached an optical density at 600 nm (OD_600_) around 5 in the enriched CDM, which exceeded the maximum cell density in complex Man-Rogosa-Sharpe (MRS) medium under the same fermentation conditions. In the first cultivation passage in CDM, which was inoculated from a working stock in complex medium, BB-46 started growing after a couple of hours, while BB-12 spent around 30 h in the lag phase. To identify which of the added nutrients are essential for each strain, in vitro omission experiments were performed in the fortified CDM, omitting one of the 15 nutrients at a time. In addition, in silico predictions regarding the strains’ prototrophy for vitamins and amino acids already included in the medium were also validated experimentally (Fig. 3b).

#### Vitamin requirements

**Fig. 4:**
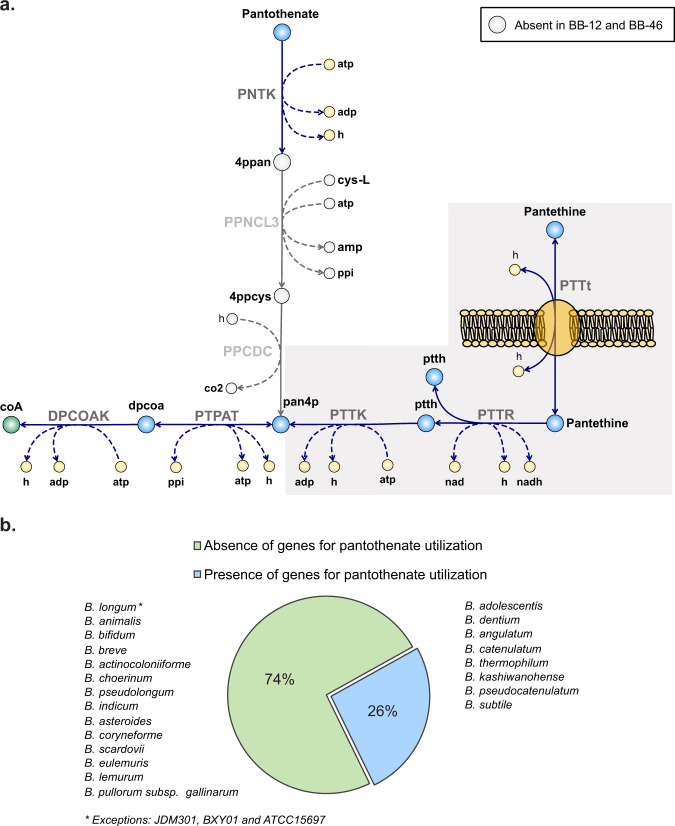
Coenzyme A biosynthesis in bifidobacteria. **a** Proposed pathway of coenzyme A (coA) biosynthesis in BB-12 and BB-46 from pantethine via pantetheine (ptth): Genes for phosphopantothenate-cysteine ligase (EC 6.3.2.5) and phosphopantothenyl cysteine decarboxylase (EC 4.1.1.36), catalyzing the conversion of phosphopantothenate (4ppan) into pantethine-4-phosphate (pan4p) through the PPNCL3 and PPCDC reactions, are absent in BB-12 and BB-46. Reactions highlighted by the gray background have been added to *i*AZ480 and *i*MS520 based on experimental evidence for pantethine utilization by BB-12 and BB-46. Added reactions include a pantethine-proton symporter (PTTt, no gene association), a pantethine reductase (PTTR, no gene association), and a pantetheine kinase (PTTK). Abbreviations of metabolite and reaction names are the same as used in the models (Supplementary Data 3 and 4). **b** Presence/absence of genes for pantothenate utilization in different *Bifidobacterium* species, based on data from publicly available genome sequences in the KEGG database.

*Vitamin B*_*5*_: Pantothenate (vitamin B_5_) was the only precursor in the generic CDM for the biosynthesis of coenzyme A (CoA), which is a crucial coenzyme in sugar catabolism and fatty acid biosynthesis. The GEMs revealed that BB-12 and BB-46 share an incomplete pathway for CoA biosynthesis from pantothenate (Fig. 4a). As described for other *Bifidobacterium* strains before, both strains lack the genes coding for phosphopantothenate-cysteine ligase (EC 6.3.2.5) and phosphopantothenoyl cysteine decarboxylase (EC 4.1.1.36). Initially, the reactions of PPNCL3 and PPCDC were added to *i*AZ480 and *i*MS520 as non-gene associated reactions, since their removal abolishes in silico growth (Supplementary Data 7). According to published genome sequences of *Bifidobacterium* strains annotated in the KEGG database, almost three-quarters of the strains lack the genes for PPNCL3 and PPCDC reactions (Fig. 4b). The presence or absence of these genes was found to be species-dependent, except for two strains of *B. longum* that deviated from other strains of the species. Previous studies reported that different precursors besides pantothenate, including S-sulfonic acid-type pantetheine related compounds, pantethine and free CoA, can be used by bifidobacteria for CoA biosynthesis, whereas the capability to use a certain precursor varies between strains^36,44–48^. Based on the network reconstructions of BB-12 and BB-46, pantethine, the stable disulfide dimer of pantetheine, was identified as potential precursor for CoA biosynthesis in the strains (Fig. 3a). Our experimental results confirmed that the strains require either pantethine or free CoA and cannot use pantothenate (Fig. 3b). Based on the findings, three additional reactions were included in *i*AZ480 and *i*MS520 to link pantethine to CoA biosynthesis via the intermediate pantetheine (Fig. 4a). The precise transport mechanism of pantethine in bifidobacteria has not been described previously, but an active transport mechanism was proposed in *Bifidobacterium breve* N4^36,49^. Therefore, a pantethine-proton symport reaction (PTTt) was included in the model without gene association. A pantethine reductase reaction (PTTR) was also included without gene association, whereas the pantetheine kinase reaction (PTTK) was associated to the gene encoding pantothenate kinase (EC 2.7.1.33), previously reported to show a broad substrate specificity including pantetheine^50^.

**Fig. 5:**
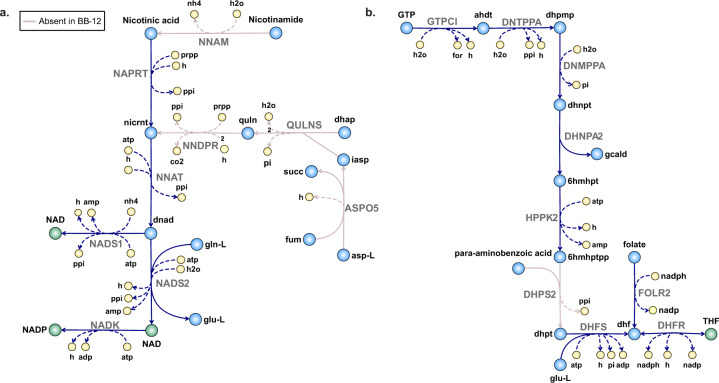
NAD(P)^+^ and tetrahydrofolate (THF) synthesis in BB-12 and BB-46. **a** Proposed pathways of NAD(P)^+^ biosynthesis in BB-12 and BB-46: In contrast to BB-46, BB-12 lacks a gene coding for nicotinamidase (EC 3.5.1.19), which catalyzes the conversion of nicotinamide into nicotinic acid (NNAM), as well as genes required for biosynthesis of nicotinate D-ribonucleotide (nicrnt) from L-aspartate (asp-L) and fumarate (fum). **b** Proposed pathways of folate biosynthesis in BB-12 and BB-46: Both strains cannot synthesize *para*-aminobenzoic acid. BB-12 further lacks a gene coding for dihydropteroate synthase (EC 2.5.1.15), catalyzing the condensation of *para*-aminobenzoic acid and 6-hydroxymethyl-dihydropterin pyrophosphate (6hmhptpp) (DHPS2). Abbreviations of metabolite and reaction names are the same as used in the models (Supplementary Data 3 and 4).

*Vitamin B*_*3*_: Nicotinic acid and nicotinamide are two forms of vitamin B_3_ that can serve as precursors for the biosynthesis of NAD(P)^+^. *i*MS520 predicted that BB-46 can synthesize NAD(P)^+^ from L-aspartate and fumarate and that it does not require any form of vitamin B_3_, although it possesses all necessary genes for utilization of nicotinic acid or nicotinamide as precursors (Fig. 5a). In agreement with the in silico predictions, BB-46 grew in CDM lacking nicotinic acid and nicotinamide (Fig. 3b). In contrast, *i*AZ480 predicted an absolute requirement for growth of BB-12 for nicotinic acid and the inability of the strain to utilize nicotinamide as an alternative precursor. Since the generic CDM contained only nicotinamide, nicotinic acid was tested as one of the potentially essential nutrients missing in the medium (Fig. 3a). The experimental results confirmed the absolute requirement of BB-12 for nicotinic acid and a lack of effect on growth of the removal of nicotinamide from the medium (Fig. 3b). Based on the KEGG database, the absence of a gene for nicotinamidase (EC 3.5.1.19) is a shared characteristic among all sequenced *B. animalis* strains, whereas all sequenced *B. longum* strains possess the gene.

*Vitamin B*_*9*_: Tetrahydrofolate (THF), the active form of folate (vitamin B_9_), is an essential cofactor for nucleic acid synthesis. The ability of *Bifidobacterium* strains to produce folate was previously reported to be strain-dependent^51–53^ and in some strains promoted by the supply of *para*-aminobenzoic acid^54^. In bacterial folate biosynthesis, *para*-aminobenzoic acid is required for the formation of dihydropteroate in the dihydropteroate synthase (EC 2.5.1.15) catalyzed reaction (DHPS2; Fig. 5b). *i*MS520 predicted a requirement of BB-46 for *para*-aminobenzoic acid in the absence of folate in the medium, whereas *i*AZ480 predicted an absolute requirement of BB-12 for folate, as it lacks a gene encoding the enzyme catalyzing the DHPS2 reaction (Fig. 3b). Two essential reactions for folate biosynthesis, namely DNTPPA and DNMPPA, were initially missing in the draft reconstructions but were added with the associated genes during the manual curation stage (Supplementary Data 7). The experimental results confirmed the absolute requirement of BB-12 for folate and the ability of BB-46 to grow in the absence of folate when *para*-aminobenzoic acid is supplied (Fig. 3b).

*Vitamin K*_*2*_: Menaquinone (vitamin K_2_) is known as an electron carrier in bacteria^55^. Previous studies indicated that some forms of vitamin K, such as menaquinone, act as growth factors for bifidobacteria^56,57^ but cannot be synthesized by representatives of the genus^58^. *i*AZ480 and *i*MS520 lack a pathway for the biosynthesis of any of the vitamin K homologs, while they suggest its potential requirement as a cofactor in some redox reactions, such as the dihydroorotic acid dehydrogenase (EC 1.3.5.2) reaction (DHORD5). Moreover, the genome sequences of BB-12 and BB-46 indicate the presence of a gene encoding NADPH-quinone oxidoreductase (EC 1.6.5.5), which requires a quinone as redox mediator. Based on these findings, commercially available menaquinone-4 was tested as one of the potentially essential nutrients missing in the generic CDM (Fig. 3a). The experimental results revealed an absolute requirement of BB-46 for menaquinone-4 (Fig. 3b). On the other hand, BB-12 showed irreproducible growth, in terms of final biomass yield and lag phase duration, in the absence of the vitamin. Further investigations are required to reveal the exact function of menaquinone in BB-12 and BB-46.

*Other B-vitamins: i*AZ480 and *i*MS520 predicted neither a requirement of BB-12 and BB-46 for pyridoxal (vitamin B_6_) nor for the vitamin-like nutrient lipoic acid. These predictions were confirmed experimentally (Fig. 3b). Due to incomplete biosynthesis pathways of riboflavin (vitamin B_2_), biotin (vitamin B_7_), thiamine (vitamin B_1_), and cobalamin (vitamin B_12_), the GEMs predicted an absolute requirement of both strains for all four vitamins. The requirement of these vitamins was, however, not validated experimentally (Fig. 3b).

Overall, the different vitamin requirements of BB-12 and BB-46 are in line with the varying abilities reported for other *Bifidobacterium* strains to synthesize and accumulate B-vitamins^51,59^.

#### Polyamine requirements

Polyamines, such as spermidine and putrescine, play a role in cellular growth and are involved in a number of metabolic and physiological processes in a variety of organisms^60^. Some *Bifidobacterium* strains were found to produce marginal amounts or take up various polyamines^61–64^. Putrescine and spermidine were, therefore, incorporated in the BOF of *i*AZ480 and *i*MS520, as part of the soluble pool. As genes for the *de-novo* biosynthesis of both compounds are missing in both strains, putrescine and spermidine were considered as essential nutrients that need to be supplied in the medium. However, our experiments revealed no requirement of BB-12 or BB-46 for either compound (Fig. 3a). Based on these results, putrescine and spermidine have either no essential function in the metabolism of the strains or they are formed using an unknown biosynthetic pathway^63^. Due to a lack of evidence, putrescine and spermidine were removed from the BOF to match the experimental results and allow in silico growth in their absence. This highlights the critical role of the BOF when determining the nutritional requirements of a strain using constraint-based modeling, since every metabolite included in the BOF must be synthesized by the strain or provided in the in silico medium. In this context, it is the presence or absence of the metabolite that is crucial rather than its exact fraction in the BOF.

#### Nucleobase requirements

It was previously reported that some *Bifidobacterium* strains require free nucleotides for growth^45,65^. Even though *i*AZ480 and *i*MS520 did not predict any such requirements, a set of nucleobases, nucleobase derivatives, and nucleobase precursors were tested as potentially essential nutrients. In agreement with model predictions, BB-12 and BB-46 grew in a CDM when omitting all these nutrients (Fig. 3a).

#### Precursors of peptidoglycan biosynthesis

The glycan moiety of peptidoglycan in the bacterial cell wall is composed of alternating *β*-1,4-linked N-acetylglucosamine and N-acetyl muramic acid residues. N-acetylglucosamine was previously suggested to be essential for growth of some *Bifidobacterium* strains^48,66^. In addition, the requirement for L-ornithine, which is a component of the interpeptide bridge in the peptidoglycan subunit of *B. longum* strains, was investigated. The results from the single omission experiments revealed that both strains neither require N-acetylglucosamine nor L-ornithine in the CDM, which matches the GEM predictions (Fig. 3a).

#### Amino acid requirements

**Fig. 6:**
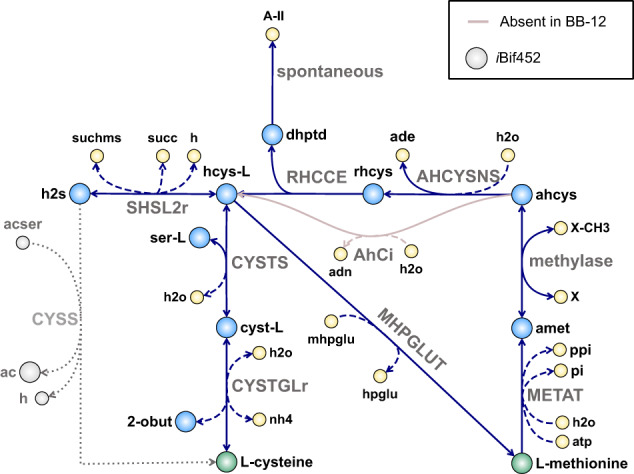
Sulfur metabolism in BB-12 and BB-46. Either cysteine or methionine can serve as the sole amino acid and sulfur source for growth of BB-12 and BB-46. Methionine is proposed to be synthesized from cysteine through the irreversible conversion of homocysteine (hcys-L) to methionine (MHPGLUT). For the synthesis of cysteine from methionine, hcys-L might be formed through the sequential formation of S-adenoysl-L-methionine (amet), S-adenosyl-L-homocysteine (ahcys), and ribosylhomocysteine (rhcys). BB-46 possesses a gene encoding adenosylhomocysteinase (EC 3.3.1.1), which might alternatively convert ahcys in hcys-L (AhCi) directly. *i*AZ480 and *i*MS520 predicted that BB-12 and BB-46 can assimilate H_2_S through its incorporation into O-succinyl-L-homoserine to generate homocysteine (SHSL2r). Alternatively, the GEM of *B. adolescentis* L2-32 suggests H_2_S assimilation via the reaction of cysteine synthase (EC 2.5.1.47, CYSS), which lacks gene association and requires an additional non-gene-associated reaction to produce acetyl-serine (acser)^24^. Abbreviations of metabolite and reaction names are the same as used in the models (Supplementary Data 3 and 4).

Bifidobacteria have previously been reported to require cysteine as an organic sulfur source^47^ due to their inability to assimilate inorganic sulfur^28,38,67^. A similar conclusion can be drawn through *i*AZ480 and *i*MS520 simulations, which also indicate that BB-12 and BB-46 are prototrophic for all other proteinogenic amino acids than cysteine. In contrast to a previous study^38^, our experimental results revealed that the requirement for cysteine can be overcome by the addition of methionine as the sole amino acid and sulfur source in the medium (Fig. 3b). According to the network reconstructions, both strains can synthesize methionine from cysteine through the direct conversion of homocysteine to methionine (MHPGLUT), but the reverse direction is not operational, since the MHPGLUT reaction is assigned as irreversible (Fig. 6). To explain our experimental results, alternative routes that allow the utilization of methionine as the sole sulfur source were evaluated. One route for the conversion of methionine to homocysteine in BB-12 and BB-46 could be through the sequential formation of S-adenosyl-L-methionine, S-adenosyl-L-homocysteine, and finally S-ribosylhomocysteine (Fig. 6). The final reaction for converting S-ribosylhomocysteine to homocysteine (i.e., RHCCE) is catalyzed by LuxS (EC 4.4.1.21), which has previously been identified in *B. longum* NCC2705 and was linked to the formation of autoinducer-2 molecules^68^. In BB-46, S-adenosyl-L-homocysteine might alternatively be directly converted into homocysteine by the adenosylhomocysteinase (EC 3.3.1.1, Fig. 6). The proposed pathways did, however, not carry any flux during in silico simulations since the production of S-adenosyl-L-homocysteine in the models was insufficient. The only reaction in the models producing S-adenosyl-L-homocysteine was the reaction of cyclopropane fatty acid synthase (EC 2.1.1.79), which is constrained by the amount of cyclopropane fatty acid required for growth. The capability to use methionine as sole sulfur source was implemented into the GEMs by adding a sink (supply) reaction for S-adenosyl-L-homocysteine (default: constrained to not carry flux). In vivo, the activity of DNA-cytosine methyltransferase (EC 2.1.1.37) may contribute to the formation of S-adenosyl-L-homocysteine. Besides cysteine and methionine, in silico predictions further suggested that BB-12 and BB-46 can use peptides as an organic sulfur source, which agrees with former studies demonstrating the ability of bifidobacteria to grow in a medium containing hydrolyzed casein instead of free amino acids^38,39^.

Previous studies suggested that sulfur-containing metabolites, such as hydrogen sulfide (H_2_S), methanethiol, or glutathione, may function as the sole sulfur source in bifidobacteria^28,38,69^. *i*AZ480 and *i*MS520 predicted that BB-12 and BB-46 can assimilate H_2_S through its incorporation into O-succinyl-L-homoserine to generate homocysteine by an O-succinyl-L-homoserine succinate-lyase (EC 2.5.1.48, SHSL2r, Fig. 6). Alternatively, the GEM of *B. adolescentis* L2-32 suggests H_2_S assimilation via cysteine synthase (EC 2.5.1.47), which lacks gene association and requires an additional non-gene-associated reaction to produce acetyl-serine^24^. Contradictory conclusions have been drawn about the presence of cysteine synthase in bifidobacteria^28,67^. Much of the ambiguity is caused by inconclusive gene annotation; the same gene is assigned to the function of cystathionine beta-synthase (EC 4.2.1.22) or cysteine synthase (EC 2.4.1.47) depending on the functional annotation source. Due to the tight safety restrictions around the laboratory use of H_2_S, we have tested the ability of an H_2_S-releasing compound, GYY4137, to serve as the sole sulfur source in the CDM. However, no growth of BB-12 or BB-46 was observed under this condition.

In contrast to previous reports^28,38^, *i*AZ480 and *i*MS520 predicted that neither glutathione nor methanethiol can serve as a sole sulfur source, as the genes encoding glutathione hydrolase and methanethiol oxidase are absent.

#### Design of an optimum chemically defined medium

The model-guided investigation of the nutritional requirements of BB-12 and BB-46 revealed that (i) both strains have an absolute requirement for pantethine; (ii) BB-12 requires nicotinic acid, but not nicotinamide; and (iii) menaquinone-4 is essential for growth of BB-46 and has a growth-promoting effect on BB-12. All three essential nutrients were identified based on the network reconstruction and in silico predictions (Fig. 3a), emphasizing the value of GEMs for medium development. Based on these findings, a complete CDM that allows optimal growth of both strains was formulated by supplementing the initial (generic) medium with pantethine, nicotinic acid, and menaquinone-4 (Table 1). The complete CDM contains some vitamins that proved not to be essential for growth of the strains (Fig. 3b), but were kept in the medium to cover possible auxotrophies in other *Bifidobacterium* strains. Furthermore, all amino acids were included in the medium, since they showed a growth-promoting effect. No CDM reported in literature for bifidobacteria contained the combination of pantethine, menaquinone-4, and nicotinic acid^38–41^, which explains their inability to sustain reproducible growth of BB-12 and BB-46 in this study for multiple subcultures. Even though early publications described pantethine as an essential growth factor for *Bifidobacterium* strains^36,44,47,48^, several newer studies on medium development for bifidobacteria have ignored this finding^39–41^. For example, a previously developed CDM for a *B. animalis* strain lacked pantethine and nicotinic acid^39^. The absence of both vitamins explains the negligible growth of the strain unless it is supplemented with acid-hydrolyzed casein^39^, which presumably provides small amounts of the essential vitamins.

**Table 1:** Composition of the newly formulated chemically defined medium for growth of BB-12 and BB-46.

Component	Concentration
Sucrose	10 [g L^−1^]
NH_4_Cl	1 [g L^−1^]
Citrate	0.6 [g L^−1^]
NaHCO_3_	0.42 [g L^−1^]
FeCl_2_ *·* 4H_2_O	6.5 [mg L^−1^]
MnCl_2_ *·* 4H_2_O	23.1 [mg L^−1^]
ZnCl_2_	0.07 [mg L^−1^]
H_3_BO_3_	0.006 [mg L^−1^]
CoCl_2_ *·* 6H_2_O	0.19 [mg L^−1^]
CuCl_2_ *·* 2H_2_O	0.002 [mg L^−1^]
NiCl_2_ *·* 6H_2_O	0.024 [mg L^−1^]
MgCl_2_ *·* 6H_2_O	400 [mg L^−1^]
CaCl_2_ *·* 2H_2_O	50 [mg L^−1^]
Cysteine-HCl	500 [mg L^−1^]
Proteinogenic amino acids but cysteine	each 40 [mg L^−1^]
K_2_HPO_4_	8.7 [g L^−1^]
KH_2_PO_4_	6.1 [g L^−1^]
*Para*-aminobenzoic acid	0.5 [mg L^−1^]
Calcium pantothenate	4 [mg L^−1^]
Pantethine	10 [mg L^−1^]
Biotin	0.2 [mg L^−1^]
Folic acid	0.2 [mg L^−1^]
Nicotinamide	0.5 [mg L^−1^]
Nicotinic acid	5 [mg L^−1^]
Pyridoxal-HCl	2 [mg L^−1^]
Riboflavin	0.5 [mg L^−1^]
Thiamine-HCl	0.5 [mg L^−1^]
Cobalamin	0.5 [mg L^−1^]
dl-6,8-Thioctic acid	0.5 [mg L^−1^]
Menaquinone-4 in 96 % EtOH	1 [mg L^−1^]
Tween^®^ 80	1 [mL L^−1^]

#### Carbohydrates utilization

**Fig. 7:**
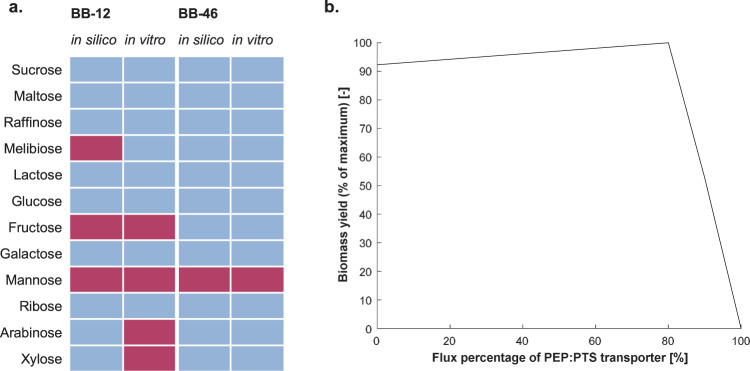
Carbohydrate utilization by BB-12 and BB-46. **a** The capability of BB-12 and BB-46 to utilize 12 different carbohydrates was tested in batch fermentations in the newly formulated chemically defined medium. Presence or absence of growth is indicated by blue and red colors, respectively. Knowledge gained from the experiments was incorporated into the models so that the model predictions agree with the in vitro results. **b** Effect of increasing glucose flux percentage through the PEP:PTS transport system on biomass yield of BB-46. 100%: flux only through the PEP:PTS transport system, 0%: transport only through the glucose proton symporter. The amino acid uptake rates were constrained to 1 mmol g_CDW_^−1^ h^−1^.

The newly formulated CDM was further used for the experimental validation of the capabilities of the GEMs to simulate growth on twelve carbohydrates, including monosaccharides (pentoses and hexoses) and oligosaccharides, as the sole carbon source (Fig. 7a). Both the transport mechanism and the pathway of the breakdown of a given carbohydrate determine the biomass yield predicted by the GEM. The model predictions were validated in batch fermentations (Fig. 7a, Supplementary Table 2). In agreement with model predictions, neither strain could grow on the newly formulated CDM in the absence of a carbohydrate. As predicted by *i*MS520, BB-46 grew on all tested carbohydrates but mannose. The predictions of *i*AZ480 agreed with the experimental results for nine out of the twelve tested carbohydrates, which is further discussed below.

*Arabinose and xylose:* In contrast to *i*AZ480 predictions, batch fermentation experiments showed that BB-12 does not grow on xylose or arabinose, whereas growth was observed on melibiose (Fig. 7a). Accordingly, the transport reactions for arabinose (non-gene associated) and xylose were removed from the model and a melibiose transport reaction was added. In *B.* *animalis* subsp. *lactis* Bl-04, an ABC transporter of raffinose was shown to act on various α-(1,6)-linked glucosides and galactosides, including melibiose^70^. Based on this finding, a melibiose transporter was added to the same genes as the raffinose ABC transporter in *i*AZ480 and *i*MS520.

*Mannose:* Both strains are unable to grow on mannose (Fig. 7a) as they lack genes for the transport and breakdown of this sugar. The capability to utilize mannose has been previously discussed for *B. longum* NCC2705, a strain closely related to BB-46^14,71^. One of the studies claimed that the strain can grow on mannose but not on arabinose^14^, which does not agree with the absence of genes for the transport and breakdown of mannose in NCC2705’s genome.

*Fructose:* Both model simulations and experimental results confirmed the inability of BB-12 to grow on fructose as the sole carbon source (Fig. 7a). This may be attributed to the lack of any fructose transporters or the presence only of a phosphoenolpyruvate phosphotransferase system (PEP:PTS) for fructose transport, as previously described in strains of *Bifidobacterium animalis* subsp. *lactis* and *Bifidobacterium bifidum*^71^. According to in silico predictions, an exclusive use of a PEP:PTS system for the transport of a hexose, such as fructose, is infeasible in bifidobacteria since the uptake of a hexose would consume the single PEP generated per hexose in the bifid shunt, making it unavailable for anabolic reactions. This may generally explain the low number of PEP-PTS transporters found in the genomes of bifidobacteria^28,69^. This prediction is in line with previous experimental and computational results, postulating that the exclusive use of a PEP:PTS transporter in the obligate heterofermentative bacterium *Leuconostoc mesenteroides* is infeasible for glucose uptake^72,73^. On the other hand, BB-46 could grow on fructose both in silico and in vitro, since it possesses the genes encoding a fructose-specific ABC transporter in its genome.

*Glucose:* The growth experiments confirmed that both strains can ferment glucose. According to *i*AZ480 and *i*MS520, the glucose uptake system differs between BB-12 and BB-46. While BB-12 takes up glucose through facilitated diffusion via the glucose permease, BB-46 exhibits two different glucose transport systems: a proton symporter and a PEP:PTS system. For *B. longum* NCC2705 it was suggested that the PEP:PTS does not contribute to glucose uptake and that the proton symporter appears to be exclusively responsible for the transport^71^. We used FBA to study how a contribution of PEP:PTS to the glucose uptake affects the biomass yield of BB-46. For this purpose, the exchange rate of glucose was set to −10 mmol g_CDW_^−1^ h^−1^ and the constraints of the transport reactions of glucose were varied between 0 and 10 mmol g_CDW_^−1^ h^−1^ in such a way that their sum always equated 10 mmol g_CDW_^−1^ h^−1^ (Fig. 7b). The most efficient transport mode was predicted to be reached when the PEP:PTS system accomplishes 80% of the glucose transport, while lower biomass yield is reached with a higher or lower contribution of the PEP:PTS system to glucose uptake. No growth is allowed if glucose is exclusively transported through PEP:PTS due to insufficient PEP, as discussed above. Similarly, FBA simulations in *L. mesenteroides* suggested that the strain takes up glucose mainly via the PEP:PTS, but a combination with a proton symporter is necessary to reach the energetic optimum^73^.

*Sucrose and raffinose:* According to both model simulations and experimental results, BB-12 and BB-46 can ferment the disaccharide sucrose and trisaccharide raffinose (Fig. 7a). Glycosyl hydrolases (GHs) involved in the raffinose and sucrose catabolism in bifidobacteria have been characterized^4,74^. Both sugars enter the bifid shunt via two different routes. Sucrose can be hydrolyzed to fructose and glucose through the sucrase enzyme (GH13 and GH32 family, according to the carbohydrate-active enzymes (CAZy) classification^75^) reaction (SUCR) or converted to fructose and glucose-1-phosphate through the sucrose phosphorylase (EC 2.4.1.7, GH13 family) reaction (SUCPHOS) using free phosphate as a phosphate donor. A higher biomass yield can be achieved via the SUCPHOS route, as it bypasses the initial ATP consumption in the bifid shunt^4^. Raffinose galactohydrolase (EC 3.2.1.22, GH27, and GH36 family) catalyzes the hydrolysis of raffinose into galactose and sucrose^74^, while raffinose fructohydrolase (EC 3.2.1.26, GH32 family) catalyzed the hydrolysis of raffinose to fructose and melibiose. A higher biomass yield is predicted with the former route. In addition, high-performance liquid chromatography (HPLC) analysis of the growth supernatant revealed the presence of galactose and sucrose, which confirms the activity of raffinose galactohydrolase in BB-46.

*Lacto-N-biose and galacto-N-biose*: BB-46 possesses all enzymes to convert lacto-N-biose and galacto-N-biose into intermediates of the bifid shunt. Previous studies confirmed the growth on lacto-N-biose in other *Bifidobacterium* strains possessing a homologous gene cluster to the one found in BB-46^76,77^. This gene cluster is, however, absent in the genome of BB-12, preventing its growth on these substrates.

Overall, the carbohydrate utilization profile of BB-12 matches that observed in a previous study using the strain^78^, whereas that of BB-46 agrees with the carbohydrate utilization profile of *B. longum* NCC2705^71^.

#### Quantitative evaluation of growth and metabolite secretion rates

To quantitatively evaluate the prediction ability of *i*AZ480 and *i*MS520, growth, sugar uptake, and fermentation end-product secretion rates by BB-12 and BB-46 were studied in the newly formulated CDM, with 10 g L^−1^ sucrose as a carbon source, in lab-scale batch fermentations. BB-12 and BB-46 showed a maximum specific growth rate of 0.45 h^−1^ and 0.35 h^−1^ (Table 2), respectively, whereas the final biomass yield of both strains was around 1.7 g_CDW_ L^−1^. A different metabolite secretion profile was observed for each strain; while BB-12 mainly secreted acetate and lactate in the exponential growth phase, the main secretion products of BB-46 were acetate, formate, and ethanol. When constraining the GEMs according to the experimentally measured sugar uptake and metabolite secretion rates (Table 2), no feasible solution could be computed by FBA. Relaxing the constraints by only including the measured sucrose uptake rate and the acetate/lactate ratio rendered FBA feasible. By fitting the maximum specific growth rates predicted by the models (i.e., BOF flux) to the experimentally measured values, growth- and non-growth-associated maintenance energy requirements could be calculated (20 mmol_ATP_ g_CDW_^−1^ and 1 mmol_ATP_ g_CDW_^−1^ h^−1^, respectively)_._ Under these conditions, model predictions largely agreed with the experimental data, except for formate secretion rate, which was higher than the experimentally measured value (Table 2). Underestimation of formate concentrations or its conversation to an undetected metabolite might be the reasons behind this deviation, which requires further investigation.

**Table 2:** Comparison of in vitro and in silico reaction rates for cultivation of BB-12 and BB-46 in the newly formulated chemically defined medium.

	BB-12 in vitro	BB-12 in silico	BB-46 in vitro	BB-46 in silico
Growth rate [h^−1^]	0.45 ± 0.0	0.44	0.35 ± 0.0	0.34
Sucrose uptake rate [mmol g_CDW_^−1^ h^−1^]	3.9 ± 0.2	3.9	2.8 ± 0.4	2.8
Acetate:lactate ratio [-]	2.5 ± 0.0	2.5	17.3 ± 0.0	17.3
Acetate secretion rate [mmol g_CDW_^−1^ h^−1^]	11.8 ± 0.7	12.0	11.9 ± 0.3	10.4
Lactate secretion rate [mmol g_CDW_^−1^ h^−1^]	4.7 ± 0.3	4.8	0.7 ± 0.0	0.6
Formate secretion rate [mmol g_CDW_^−1^ h^−1^]	0.3 ± 0.0	3.3	4.0 ± 0.2	5.8
Succinate secretion rate [mmol g_CDW_^−1^ h^−1^]	0.2 ± 0.0	0.2	0.2 ± 0.0	0.1
Ethanol secretion rate [mmol g_CDW_^−1^ h^−1^]	–	0.4	2.5 ± 0.3	1.7
Carbon recovery excluding biomass formation [%]	82	–	106	–

The sucrose uptake rate and the acetate:lactate ratio were constrained to the experimentally determined values. Amino acid uptake rates were constrained to 1 mmol g_CDW_^−1^ h^−1^. The carbon recovery was calculated based on the specific rates determined during the exponential growth phase and does not include biomass formation. In vitro reaction rates are given as means of three replicates (*n* = 3) ± standard deviations.

In conclusion, we have generated a complete genome sequence of the probiotic bacterium *B. longum* subsp. *longum* BB-46 and created a high-quality, experimentally validated genome-scale metabolic model for the strain as well as for another industrially and clinically important probiotic strain, *B. animalis* subsp. *lactis* BB-12. We have interrogated the models to probe the metabolic landscape of the strains and identified their essential nutritional requirements. Insights obtained from the models were used to develop a CDM that supports the optimal growth of both strains in the same manner as complex culture medium. We highlighted the value of careful curation of network reconstructions if organism-specific GEMs are to be obtained, as well as the importance of properly defining the BOF components to obtain reliable predictions that can guide medium design. Together with the GEMs, the newly formulated CDM provides an essential tool to further explore the metabolism and physiology of the two species, investigate the mechanisms underlying their health-promoting effects and guide the optimization of their industrial production processes. The medium can also serve as a pharma-grade alternative to complex industrial media for the production of the strains, allowing the compliance with stricter pharmaceutical standards, e.g., in live biotherapeutic applications. Furthermore, the experimentally validated GEMs generated in this study offer useful templates for the reconstruction of additional high-quality GEMs of other *Bifidobacterium* species, which can significantly reduce the time and effort required for their manual curation and refinement.

## Methods

### Bacterial strains

The strains *B. animalis* subsp. *lactis* BB-12^®^ and *B. longum* subsp. *longum* BB-46 were obtained from the Chr. Hansen Culture Collection.

### Cultivation conditions

BB-12 and BB-46 were routinely maintained as glycerol stocks prepared as follows. Strains were cultivated by two consecutive passages in 50 mL MRS medium containing 10 g L^−1^ sucrose as carbon source, under anaerobic conditions in crimp-top serum bottles. To attain anaerobic conditions the headspace of serum bottles containing MRS was flushed with 100% N_2_ for 20 min under agitation (through magnetic stirrer) before sterilization, and cysteine-HCl was added to the medium as a reducing agent at a final concentration of 0.5 g L^−1^ prior to inoculation. Temperature was maintained at 37 °C and agitation (magnetic stirring) was applied at 400–500 rpm to keep the cells in suspension. Exponentially growing cells of the first passage were used to initiate a second cultivation at an OD_600_ of 0.05. The cells were grown until exponential growth phase (OD_600_ about 1) and stored in 20% (v/v) glycerol at −80 °C.

Batch fermentations of BB-46 and BB-12 were performed under anaerobic conditions in crimp-top serum bottles with a working volume of 25–200 mL using media indicated in the individual experiments, without pH control (initial pH 6.5). Anaerobiosis was attained by flushing the headspace of serum bottles with a gas mixture of 80% (v/v) N_2_ and 20% (v/v) CO_2_ for 20 min under agitation (magnetic stirring) before medium sterilization. In addition, cysteine-HCl (0.5 g L^−1^) was added to the medium as reducing agent prior to inoculation. Temperature and agitation (magnetic stirring) were maintained at 37 °C and 400–500 rpm, respectively. Batch cultivations were initiated at an OD_600_ of 0.05 by addition of a preculture in the exponential growth phase (OD_600_ about 1). To adjust bacteria to the culture medium and decrease culture variability, exponentially growing cells were transferred at least twice in the same cultivation medium before the main culture. Fermentation experiments with quantitative analysis of external metabolite concentrations were performed in triplicates. Nutrient omission experiments were run in duplicates.

### Media composition

The generic CDM used in this study contained the following nutrients: 10 g L^−1^ sucrose, 1 g L^− 1^ NH_4_Cl, 0.6 g L^−1^ citrate, 0.42 g L^−1^ NaHCO_3_, 6.5 mg L^−1^ FeCl_2_ *·* 4H_2_O, 23.1 mg L^−1^ MnCl_2_ *·* 4H_2_O, 0.07 mg L^−1^ ZnCl_2_, 0.006 mg L^−1^ H_3_BO_3_, 0.19 mg L^−1^ CoCl_2_ *·* 6H_2_O, 0.002 mg L^−1^ CuCl_2_ *·* 2H_2_O, 0.024 mg L^−1^ NiCl_2_ *·* 6H_2_O, 400 mg L^−1^ MgCl_2_ *·* 6H_2_O, 50 mg L^−1^ CaCl_2_ *·* 2H_2_O, 500 mg L^−1^ cysteine-HCl, 40 mg L^−1^ of L-alanine, L-arginine, L-asparagine, L-aspartic acid, L-glutamine, L-glutamic acid, glycine, L-histidine, L-isoleucine, L-leucine, L-lysine, L-methionine, L-phenylalanine, L-proline, L-serine, DL-threonine, L-tryptophan, L-tyrosine and L-valine, 8.7 g L^−1^ K_2_HPO_4_, 6.1 g L^−1^ KH_2_PO_4_, 0.5 mg L^−1^ *para*-aminobenzoic acid, 0.2 mg L^−1^ biotin, 4 mg L^−1^ calcium pantothenate, 0.2 mg L^−1^ folic acid, 0.5 mg L^−1^ nicotinamide, 2 mg L^−1^ pyridoxal-HCl, 0.5 mg L^−1^ cobalamin, 0.5 mg L^−1^ riboflavin, 0.5 mg L^−1^ thiamine-HCl, 0.5 mg L^−1^ dl-6,8-thioctic acid, and 1 mL L^−1^ Tween^®^ 80. Following the recommendations of the supplier, stock solutions of individual and sets of medium components were either sterile filtered using 0.22 µm filter or autoclaved for 10–15 min at 121 °C before being combined to the final concentrations. For the determination of the macromolecular composition of the cells, a semi-chemically defined medium was used, whose composition was similar to that of the generic CDM and supplemented with 0.5 g L^−1^ yeast extract (Supplementary Note 1).

### Analytical methods

To monitor cellular growth throughout the fermentations, the OD_600_ of the culture broth was regularly measured using an Eppendorf BioPhotometer (Eppendorf, Hamburg, Germany). The CDW was determined in triplicates using 10 mL aliquots of cell suspension harvested in the exponential growth phase. The cell suspension was filtered through a pre-dried membrane filter (0.22 µm) and washed three times with equal amount of Milli-Q water and allowed to dry to a constant weight, before determining the CDW. A linear correlation between OD_600_ and CDW was established, with one unit of OD_600_ corresponding to 0.30 g_CDW_ L^−1^ for BB-12 and 0.32 g_CDW_ L^−1^ for BB-46. The concentrations of fermentation end products in the culture broth were determined on a HPLC system (LC-4000, Jasco Inc-Easton, MD, USA) equipped with both a UV (210 nm) and RI detectors using a Rezex ROA H^+^ (8%) ion-exclusion column (Phenomenex, Torrance, CA, USA) at 80 °C, with 5 mM H_2_SO_4_ as mobile phase and a flow rate of 0.6 mL min^−1^. Sucrose concentrations were determined by high-performance anion-exchange chromatography with pulsed amperometric detection (HPAE-PAD) on an Ultimate 5000 system (Thermo Fisher Scientific, Sunnyvale, CA, USA) using Dionex CarboPac PA1 guard and analytical columns (Thermo Fisher Scientific, Sunnyvale, CA, USA). The separation was performed under isocratic conditions at 30 °C with 100 mM NaOH as eluent at a flow rate of 1 mL min^−1^.

### Genome sequencing and assembly

The complete genome of BB-12 was sequenced in a previous study^79^ and was retrieved from NCBI GenBank (Accession no. CP001853.2). The genome of BB-46 was sequenced in this study, using both Illumina short-read and Oxford Nanopore Technologies (ONT) long-read sequencing platforms. For short-read sequencing, genomic DNA (gDNA) was extracted from an exponentially growing culture (OD_600_ = 1) with the DNeasy Blood and Tissue kit on a QiaCube system (Qiagen, Hilden, Germany), following the manufacturer’s protocol. The DNA was fragmented using a BioRuptor sonication device (Diagenode Inc., Denville, NJ, USA) with a target insert size of 800 bp. A gDNA library was generated using the KAPA HyperPlus Kit (Roche, Basel, Switzerland), according to the manufacturer’s protocol. Sequencing was performed on an Illumina MiSeq system using a MiSeq Reagent Kit v3 (Illumina Inc., San Diego, USA), with a paired-end protocol and a read length of 2 × 300 bp. For long-read sequencing, gDNA extraction, library preparation, and sequencing were performed at GenXone Sequencing (Suchy Las, Poland). Briefly, bacterial cell pellets were harvested by centrifugation from overnight cultures (OD_600_ = 1.5). gDNA was extracted by Genomic Micro AX Bacteria Gravity Kit (A&A Biotechnology, Gdynia, Poland), following the manufacture’s protocol. The sequencing library was prepared using a Rapid Barcoding Kit (SQK-RBK004; ONT, Oxford, UK) and was subjected to an additional purification and concentration step using AMPure XP beads (Beckman Coulter, Brea, CA, USA). Sequencing was performed on a MinION flow cell (R9.4.1 FLO-MIN106; ONT, Oxford, UK). A combination of the short and long sequencing reads was used to create a hybrid genome assembly for BB-46 with Unicycler^80^. The assembly was run under Unicycler’s ‘conservative’ mode in order to minimize sequence misassembly. Open reading frames (ORFs) in the assembled genome were identified and functionally annotated using the NCBI Prokaryotic Genome Annotation Pipeline (PGAP).

### Genome-scale metabolic network reconstruction

The genome-scale metabolic networks of BB-12 and BB-46 were reconstructed following a semi-automated approach (Supplementary Data 7). A draft network reconstruction of BB-12 was first generated using Model SEED^81^. The Model SEED reaction identifiers were converted to BiGG reaction identifiers^82^ using the MetaNetX/MNXref namespace^83^ and the corresponding reaction descriptions and reaction formulas were retrieved from the BiGG database. Metabolite identifiers, charged formulas and charges were also retrieved from the BiGG database. The draft reconstruction was then subjected to manual curation in a pathway-by-pathway manner. In this process, an improved functional genome annotation of BB-12 that integrates information from multiple databases was retrieved from the Integrated Microbial Genomes system (https://img.jgi.doe.gov/)^84^ and was used to refine the RAST annotations^85^, originally used for the draft reconstruction by Model SEED. Additional reactions were included in the draft reconstruction from the BiGG database^82^ when biochemical, physiological, or genomic evidence on their presence in BB-12 existed. Besides, reactions originally included in the draft reconstruction were removed if their presence in the strain could not be verified. During the curation process, we conveyed available literature on the strain and closely related microorganisms as well as data compiled from the online databases KEGG^29^, TransportDB^86^, and BioCyc^87^. GPR associations for all reactions were verified and adjusted, when necessary; some reactions were incorporated in the reconstruction without GPR when experimental evidence on the presence of the corresponding functions in BB-12 existed.

Following the conversion of the network reconstruction to a mathematical model and the process of model debugging and refinement, which are described in detail below, the curated metabolic network of BB-12 was used as a template to automatically generate a draft network reconstruction for BB-46. This draft reconstruction was created based on protein sequence homology of translated ORFs, using MetaDraft v0.8.1^88^. Non-gene associated reactions from BB-12 GEM were also carried over to BB-46 reconstruction and re-examined to verify their relevance in the strain. The draft reconstruction of BB-46 was then manually curated following the same approach described above. In addition, candidate reactions associated with genes of BB-46 without homologs in BB-12 genome were identified using an additional set of manually curated GEMs as templates. These included *Escherichia coli i*JO1366^89^, *Mycobacterium tuberculosis iNJ*661^90^, *Lactococcus lactis* MG1363^91^, *Clostridium ljungdahlii* iHN637^92^, *Streptococcus thermophilus* LMG18311^32^, *Geobacter metallireducens* iAF987^93^ and *Staphylococcus aureus i*SB619^94^. Candidate reactions identified from the additional template models were retained in the reconstruction of BB-46 based on available evidence. For those additional reactions that were retained, a bidirectional BLAST of the associated protein sequence(s) was performed against the translated ORFs in the BB-12 genome. If a homolog was found, the corresponding gene was included in the BB-12 reconstruction, resulting in the incorporation of additional reactions or the assignment of additional genes to previously incorporated reactions in the BB-12 GEM.

To visualize different pathways or subsystems in the metabolic network, the Omix Visualization software version 1.8 was used^95^.

### Biomass composition

For the formulation of a *Bifidobacterium*-specific BOF^96,97^, CDW of BB-12 was divided into the main categories of protein, DNA, RNA, carbohydrates, cell wall components (peptidoglycan, capsular polysaccharide, and lipoteichoic acid), lipids, and inorganic ions and metabolites. The total protein content of the BB-12 cells was determined by the Biuret method, with bovine serum albumin as a standard^98^. Total genomic DNA was quantitatively extracted using the Easy-DNA kit (Invitrogen, CA, USA), according to the manufacturer’s protocol and DNA concentration was measured spectrophotometrically at 260 nm using a NanoDrop 2000 (Thermo Scientific). The total cellular RNA content was quantified by the KOH/UV method^99^. Total carbohydrate content of BB-12 cells (capsular polysaccharide and free sugars in addition to sugar residues in peptidoglycan and lipoteichoic acids) was determined using the phenol-sulfuric acid method^98^. The remaining biomass fraction was assigned to cell wall components, lipids, inorganic ions, and soluble metabolites based on average ratios in other bacteria.

The average amino acid composition of the protein fraction of the CDW was estimated from the codon usage frequency for each amino acid, as calculated from all protein-encoding genes in BB-12 genome. The structure of the capsular polysaccharide subunit was considered to be the same as in *B. animalis* subsp. *lactis* LKM512^100^. The structure of the peptidoglycan subunit in each strain was deduced from their cell wall type, which is A3α for *B. animalis* subsp*. lactis*^101^ and A3β for *B. longum*^102^. The cellular fatty acid profile was adapted from that of *Bifidobacterium bifidum* var. *pennsylvanicus*^43^. Phospholipids were divided into diphosphatidylglycerol (cardiolipin), phosphatidylglycerol, compound 15, and compound 17, as previously described^43^. Teichoic acids were considered to be mainly glycerol-type teichoic acid associated with membrane lipids, i.e., lipoteichoic acid, as was previously reported for *B. bifidum* var. *pennsylvanicus*^43^. The concentrations of different inorganic ions as well as metabolites and cofactors in the soluble pool were adapted from the *i*JO1366^89^ and *i*AF1260 GEMs of *E. coli*^103^.

### In silico network evaluation

The refined metabolic network reconstructions of BB-12 and BB-46 were converted to a mathematical format by parsing the stoichiometric coefficients of the curated reactions using the COnstraint-Based Reconstruction and Analysis (COBRA) Toolbox version 2.17.1 within MATLAB (R2015b; MathWorks)^104^. FBA^34^, which seeks an optimal solution by optimizing an objective function through the use of linear programming (LP) methods, was employed for network evaluation. The FBA-based function GapFind^105^ in the COBRA Toolbox was used for evaluating and debugging the genome-scale metabolic networks, with Gurobi (Gurobi Optimization, LLC) as an LP solver. The mass and charge balances of all reactions were checked, and unbalanced reactions were adjusted by consulting biochemical reaction databases. The quality of the final models were evaluated using MEMOTE, a software that runs a standardized set of metabolic model test^27^. The GEMs were further evaluated by comparing simulation results with known growth characteristics of bifidobacteria using FBA.

### Determination of nutritional requirements

#### Essential nutritional requirements

Following the initial model debugging and refinements, the individual GEMs were used to identify the essential nutritional requirements of each strain through in silico single omission experiments. For this, the uptake rate was set to zero for one nutrient a time before optimizing for biomass formation using FBA. If no growth was predicted, the nutrient was considered essential. In addition, literature on nutritional requirements of closely related *Bifidobacterium* strains was consulted. Following this approach, several candidate nutrients, which could be essential for growth of the strains but were missing in the generic CDM, were identified. For the formulation of a CDM supporting growth of BB-12 and BB-46, all missing nutrients were added simultaneously to the generic CDM. The formulated CDM was tested for its ability to sustain growth of BB-12 and BB-46 in batch fermentations for three successive subcultures. Growth in complex MRS medium with 10 g L^−1^ sucrose as carbon source and 0.5 g L^−1^ cysteine as reducing agent was used as a positive control. To identify which of the added nutrients are essential for each strain, in vitro omission experiments were performed in the fortified CDM, omitting one nutrient at a time.

#### Vitamin and amino acid requirements

In silico predictions regarding the strains’ prototrophy for vitamins and amino acids already included in the generic CDM were also validated experimentally. An omitted nutrient was considered to be dispensable if the strain reached a similar final OD_600_ over two passages as in the newly formulated CDM containing all amino acids and vitamins (positive control). When removing cysteine from the medium, 0.5 g L^−1^ L-ascorbic acid was added as a reducing agent, and 0.4 g L^−1^ methionine was added as a potential alternative sulfur source.

#### Carbohydrate utilization

The GEMs were used to study the ability of both strains to use different carbohydrates as the sole carbon and energy source. FBA results were validated against experimental data from batch fermentations in the newly formulated CDM containing different carbohydrates at a concentration of 10 g L^−1^. The inoculum was grown for two passages in the CDM with sucrose as carbon source. Cellular growth was assessed by measuring OD_600_ after incubation for a maximum of 70 h. K﻿nowledge gained throughout the study was used to further refine the genome-scale metabolic networks in an iterative approach.

### Maintenance energy requirements and model validation

The ATP requirements for growth- and non-growth associated maintenance were initially set to 39.31 mmol_ATP_ g_CDW_^−1^ and 5 mmol_ATP_ g_CDW_^−1^ h^−1^, respectively, based on experimentally determined values in the Gram-positive, anaerobic bacterium *Caldicellulosiruptor saccharolyticus*^106^.

Following the model-guided development of a CDM, the GEM predictions were validated against data from batch fermentations in the newly developed medium. Nutrient uptake rates in the GEMs were constrained based on the composition of the CDM and the specific uptake and secretions rates calculated from measurements in the exponential growth phase of the fermentations. The specific rate of a metabolite was determined as the product of the specific growth rate (h^−1^) and the regression slope for the relationship of metabolite concentration (in mmol L^−1^) versus the biomass concentration (in g_CDW_ L^−1^) in the exponential growth phase. Growth and non-growth-associated maintenance requirements in each GEM were adjusted by fitting the predicted maximum specific rate to the experimentally determined value under the same condition.

## Supplementary information

Supplementary Information

Supplementary Data 1

Supplementary Data 2

Supplementary Data 3

Supplementary Data 4

Supplementary Data 5

Supplementary Data 6

Supplementary Data 7

## Data Availability

The authors declare that all the data supporting the work are available within the paper and its Supplementary Information.

## Code availability

All programs and functions used are freely available and are referred to in the Method section.
