# Supplementary material for: Identifying the essential nutritional requirements of the probiotic bacteria Bifidobacterium animalis and Bifidobacterium longum through genome-scale modeling
Source: NPJ Syst Biol Appl. 2021 Dec 9;7:47. doi: 10.1038/s41540-021-00207-4 (PMC8660834; doi:10.1038/s41540-021-00207-4)
Supplement: Supplementary file 1 — Supplementary Information [file 41540_2021_207_MOESM1_ESM.pdf]

Supplementary Information

Identifying the essential nutritional requirements of the probiotic bacteria *Bifidobacterium animalis* and *Bifidobacterium longum* through genome-scale modeling

Marie Schöpping[1,2], Paula Gaspar[1], Ana Rute Neves[1,3], Carl Johan Franzén[2], Ahmad A. Zeidan[1,*]

[1]Systems Biology, Discovery, Chr. Hansen A/S, 2970 Hørsholm, Denmark

[2]Division of Industrial Biotechnology, Department of Biology and Biological Engineering, Chalmers University of Technology, 41296 Gothenburg, Sweden

[3]Current address: Arla Foods Ingredients Group P/S, 6920 Videbæk, Denmark

*Corresponding author, DKAHZE@chr-hansen.com

List of Supplementary Information

**Supplementary Table 1:**	Sequencing statistics and other relevant characteristics of the genome sequence of BB-46
**Supplementary Data 1:**	Memote report for *i*AZ480 (HTML format)
**Supplementary Data 2:**	Memote report for *i*MS520 (HTML format)
**Supplementary Data 3:**	*i*AZ480 in Excel format
**Supplementary Data 4:**	*i*MS520 in Excel format
**Supplementary Data 5:**	*i*AZ480 in SBML format
**Supplementary Data 6:**	*i*MS520 in SBML format
**Supplementary Data 7:**	Overview of draft reconstruction, manual curation and refinement of *i*AZ480 and *i*MS520 (Excel format)
**Supplementary Note 1:**	Formulation of a *Bifidobacterium*-specific biomass objective function
**Supplementary Note 2:**	Type III extreme cycles detected in *i*AZ480 and *i*MS520
**Supplementary Note 3:**	Examples of gap filling
**Supplementary Table 2:**	Carbohydrate utilization profiles of BB-46 and BB-12

Supplementary Table 1: Sequencing statistics and other relevant characteristics of the genome sequence of BB-46.

**Assembly size**	2,385,558 bp
**N50**[1)]	2,385,558 bp
**L50**[2)]	1
**GC%**	60.33%
**Coverage (short reads)**	136x
**Coverage (long reads)**	965x
**Coverage (total)**	1101x

[1)] Half of the genome sequence is in contigs larger than or equal the N50 contig size
[2)] Minimum number of contigs accounting for at least half of the bases of the assembly.

Supplementary Note 1: Formulation of a *Bifidobacterium*-specific biomass objective function

Abstract

The current report describes the methods used to generate a biochemical equation for the biosynthesis of *Bifidobacterium animalis* ssp. *lactis* BB-12 biomass. The equation gives a general idea about the average biochemical composition of BB-12 cells and is used as a biomass objective function (BOF) for genome-scale modeling of BB-12 and BB-46 using constraint-based methods.

Materials and Methods

Culture conditions

BB-12 from a frozen stock culture was streaked onto an MRS agar plate and incubated overnight inside an anaerobic jar at 37˚C. A single colony was transferred into liquid MRS medium and incubated overnight at 37˚C. The cells were then passed three times on a semi-chemically defined medium (SCDM) supplemented with 0.5 g L^{-1} of yeast extract (Table 1). The SCDM was prepared and inoculated under strict anaerobic conditions. The third subculture was used to inoculated 150 mL of the same medium for collecting BB-12 biomass for further analyses. Cells were harvested in mid-late exponential growth phase (OD_{600} = 0.82) and 10-mL aliquots were washed by centrifugation (8000 x g; 5 min) using cold phosphate-buffered saline (pH 7.4). Washed cells were stored at -20ºC until analyzed.

Table 1. Composition of the SCDM used for growing BB-12. The medium was supplemented with 0.5 g/L yeast extract.

Component	g/L
Sucrose	10
NH4Cl	1
K_2HPO_4	3.5
KH_2PO_4	1.5
$NaHCO_3$	0.42
$MgCL_2·6H_2O$	0.4
$MnCl_2·4H_2O$	0.023
$FeCl_2·4H_2O$	0.005
$CaCl_2·2H_2O$	0.05
SL-10 Trace Elements (1000x)	1 mL
Wolf's Vitamin Solution (1000x)	10 mL
L-Alanine	0.04
L-Arginine·HCl	0.04
L-Aspargine·H_2O	0.04
L-Aspartic acid	0.04
L-Glutamic acid	0.04
Glycine	0.04
L-Histidine·HCl·H_2O	0.04
L-Leucine	0.04

L-Lysine·HCl	0.04
L-Methionine	0.04
L-Phenylalanine	0.04
L-Proline	0.04
L-Serine	0.04
DL-Threonine	0.04
L-Tryptophan	0.04
L-Tyrosine·Na	0.04
L-Valine	0.04
L-Cysteine·HCl	0.5
Tween 80	1 mL
Resazurine	0.001

Table 2. Composition of SL-10 Trace Elements (1000x).

Component	Quantity/L
HCl (25%, 7.7 M)	10 mL
$FeCl_2 \cdot 4H_2O$	1.5 g
$ZnCl_2$	0.07 g
$MnCl_2$. $4H_2O$	0.1 g
H_3BO_3	0.006 g
$CoCl_2.6H_2O$	0.19 g
$CuCl_2.2H_2O$	0.002 g
$NiCl_2.6H_2O$	0.024 g
Distilled water	990 mL

Table 3. Composition of Wolf's Vitamin Solution (1000x).

Component	mg/L
Biotin	20
Folic acid	20
Pyridoxine-HCl	100
Riboflavin	40
Thiamine-HCl	50
Nicotinic acid	50
Vitamin B_{12}	1
p-Aminobenzoic acid	50
dl-Ca-pantothenate	400
dl-6,8-Thioctic acid	50

Analytical methods and calculations

See main article.

Results

Macromolecular composition

The macromolecular composition of BB-12 used to construct the BOF is presented in the table below. Values of protein, DNA, RNA and carbohydrate fractions are averages of three independent determinations ± standard deviation.

Table 4. Macromolecular composition of BB-12. CDW: cell dry weight.

Component	Weight % (g/g_{CDW})
Protein*	52.2 ± 0.6
DNA	3.77 ± 1.0
RNA	4.9 ± 0.5
Carbohydrate**	11.5 ± 0.9
Peptidoglycan	10.0
Lipoteichoic acid	2.5
Lipid	12.0
Inorganic ions	1.0
Soluble pool	3.5

*This protein content is excluding the peptide fraction of peptidoglycan. The total protein content of BB-12 cells measured experimentally, including peptidoglycan peptides, was 57.5%.

**10% of the total carbohydrate content was allocated to capsular polysaccharide

Amino acid composition

The molar fraction of each amino acid in the total protein content of BB-12 biomass was calculated based on the codon usage frequency in all protein-encoding genes in the genome.

Table 5. Amino acid composition of protein content of BB-12. MW: molecular weight. CDW: cell dry weight.

Amino acid	Abbreviation	Molar fraction	MW (g/mol)*	mmol/g_{CDW}
L-Alanine	ala-L	0.106	71.079	0.506247
L-Arginine	arg-L	0.062	157.197	0.294202
L-Asparagine	asn-L	0.035	114.104	0.166414
L-Aspartate	asp-L	0.065	114.080	0.310305
L-Cysteine	cys-L	0.010	103.145	0.048356
L-Glutamine	gln-L	0.036	128.131	0.173330
L-Glutamate	glu-L	0.061	128.107	0.289902
Glycine	gly	0.078	57.052	0.370865
L-Histidine	his-L	0.024	137.142	0.116536
L-isoleucine	ile-L	0.054	113.160	0.258318
L-Leucine	leu-L	0.088	113.160	0.419257
L-Lysine	lys-L	0.037	129.183	0.174719
L-Methionine	met-L	0.027	131.199	0.130209
L-Phenylalanine	phe-L	0.035	147.177	0.166992
L-Proline	pro-L	0.047	97.117	0.221918
L-Serine	ser-L	0.057	87.078	0.273745
L-Threonine	thr-L	0.058	101.105	0.277573
L-Tryptophan	trp-L	0.013	186.214	0.062332
L-Tyrosine	tyr-L	0.027	163.176	0.128981
L-Valine	val-L	0.079	99.133	0.374916
Average MW			114.852	

* The MW of H_2O was subtracted from that of each amino acid to represent the loss of H_2O occurring during peptide bond formation.

Nucleotide composition

The average nucleotide composition of DNA, as deduced from the genome sequence of BB-12, is presented in the table below.

Table 6. Average nucleotide composition of DNA of BB-12. MW: molecular weight. CDW: cell dry weight.

DNA Component	Abbreviation	Molar fraction	MW (g/mol)	$mmol/g_{CDW}$
dATP	datp	0.198	312.202	0.0244
dCTP	dctp	0.303	286.16	0.0374
dGTP	dgtp	0.303	328.201	0.0374
dTTP	dttp	0.198	303.187	0.0244
Average MW			321.410	

Total RNA content of BB-12 cells was divided into rRNA (0.75), tRNA (0.2) and mRNA (0.05). The average nucleotide composition of rRNA and tRNA was calculated based on the sequence of the corresponding genes. Average nucleotide composition of mRNA was calculated based on the sequences of all protein-encoding genes in the genome.

Table 7. Average nucleotide composition of RNA of BB-12. MW: molecular weight. CDW: cell dry weight.

RNA Component	Abbreviation	Molar fraction	MW (g/mol)	$mmol/g_{CDW}$
ATP	atp	0.264	328.201	0.0408
CTP	ctp	0.340	304.175	0.0527
GTP	gtp	0.193	344.2	0.0299
UTP	utp	0.203	305.159	0.0314
Average MW			321.410	

Cell wall composition

The peptidoglycan type of BB-12 cell wall was considered to be the same as in *B. animalis* ssp. *lactis* DSM10104, which is A3α, L-Lys(Orn) — L-Ala(Ser) — L-Ala_2[1]. Therefore, the peptidoglycan subunit consists of N-acetylglucosamine, N-acetylmuramic acid, D-glutamate, L-alanine, L-lysine, D-glutamate and D-alanine in a 1:1:1:4:1:1 ratio.

Table 8. Biomass-specific peptidoglycan concentration used for the formulation of the biomass objective function of BB-12. MW: molecular weight. CDW: cell dry weight.

**Component**	**Abbreviation**	**MW (g/mol)**	**mmol/g_{CDW}**
Peptidoglycan subunit	peptido_BIF	1162.215	0.0860

The peptidoglycan type of *B. longum* subsp. *longum* cell wall is A3-β, L-Orn — L-Ser — L-Ala — L-Thr — L-Ala[2]. Thus, the peptidoglycan subunit consists of N-acetylglucosamine, N-acetylmuramic acid, L-alanine, L-ornithine, L-serine, D-threonine and D-alanine in a 1:1:3:1:1:1:1 ratio.

Table 9. Biomass-specific peptidoglycan concentration used for the formulation of the biomass objective function of BB-46. MW: molecular weight. CDW: cell dry weight.

**Component**	**Abbreviation**	**MW (g/mol)**	**mmol/g_{CDW}**
Peptidoglycan subunit	peptido_BL	1265.292	0.0790

Lipid composition

The fatty acid profile of bifidobacteria lipid fraction was adapted from Bezkorovainy & Miller-Catchpole (1989)[3].

Table 10. Fatty acid profile used for the formulation of the biomass objective functions. MW: molecular weight.

Fatty acid	Normalized content % (w/w)	MW (g/mol)	mol fraction
14:0	3.37	227.36	0.0281
Iso 14:0	1.09	227.36	0.009
16:0	22.77	255.42	0.213
16:1	5.25	253.406	0.049
18:0	22.20	283.47	0.230
18:1	38.12	281.46	0.393
18:2	1.78	279.444	0.018
Cyclo 19:0	2.87	295.5	0.031
Average MW		**272.512**	

The lipid fraction was considered to consist mainly of phospholipids. Type and factional distribution of these phospholipids were adapted from Bezkorovainy & Miller-Catchpole (1989)[3]. The molar fractions of different fatty acids calculated above were used to create an 'average' fatty acid to be incorporated into *Bifidobacterium*-specific phospholipids.

Table 21. Lipid composition used for the formulation of the biomass objective functions. MW: molecular weight. CDW: cell dry weight.

Component	Abbreviation	mol fraction	MW (g/mol)	mmol/g_{CDW}
Cardiolipin	clpn_BIF	0.4253	1420.400	0.047424
Phosphatidylglycerols, different types	pglyc_BIF	0.2725	756.155	0.030386
Compound 15	cpd15_BIF	0.1954	918.4094	0.021788
Compound 17	cpd17_BIF	0.1340	645.8397	0.014942
Average MW			935.1	

Teichoic acids

The type and composition of teichoic acid was adapted from that of *B. bifidum* var. *pennsylvanicus*, which contains mainly glycerol-type lipoteichoic acids[3,4]. The composition is detailed in the table below.

Table 12. Teichoic acid compositions used for the formulation of the biomass objective functions. CDW: cell dry weight.

Component	µmol/mg lipoteichoic acid	Monomers in lipoteichoic acid molecule	mmol/g_{CDW}
D-Galactose	1.33	17	
D-Glucose	1.24	17	
Glycerol	1.03	13	
Phosphate	1.05	13	
Fatty acids	0.31	4	
Palmitic acid	31.9		
Octadecaneoic acid	39.8		
Stearic acid	9.6		
Myristic acid	9.3		
**Lipoteichoic acid (lipota_BIF)**			**0.002931**

An average fatty acid MWt was calculated based on the proportion of each of the 4 fatty acids in lipoteichoic acids. Based on the above information, the average molecular formula of lipoteichoic acids (abbreviated 'lipota_BIF') is $C_{307}H_{546}O_{243}P_{13}$, which was used to calculate the molar fraction in CDW.

Inorganic ions

Inorganic acid composition was adapted from the *i*AF1260 genome-scale model of *E. coli*[5].

Table 13. Inorganic acid composition used for the formulation of the biomass objective functions. MW: molecular weight. CDW: cell dry weight.

Ion	Abbreviation	mol fraction	MW (g/mol)	mmol/g_{CDW}
K	k	0.7353	38.9637	0.175525
NH4	nh4	0.0490	18.039	0.011702
Mn	mg2	0.0327	23.985	0.007801
Ca	ca2	0.0196	39.9626	0.004681
Fe(+2)	fe2	0.0294	55.9349	0.007021
Fe(+3)	fe3	0.0294	55.9349	0.007021
Cu	cu2	0.0131	63.546	0.003120
Mn	mn2	0.0131	54.938	0.003120
Molybdate	mobd	0.0131	159.94	0.003120
Zn	zn2	0.0131	63.9291	0.003120
Co(+2)	cobalt2	0.0131	58.9332	0.003120
Cl	cl	0.0196	34.9689	0.004681
Inorganic phosphate	pi	0.0163	95.978	0.003901

Soluble pool

The metabolites and their fractional distribution in the soluble pool were adapted from the *i*AF1260 and *i*JO1366 genome-scale models of *E. coli*[5,6]. Metabolites that are included in the *E. coli* model but had no complete biosynthetic pathway in BB-12 and BB-46 were not available in the defined growth medium of BB-12 were not accounted for.

Table 14. Soluble pool composition used for the formulation of the biomass objective functions. CDW: cell dry weight.

Metabolite	Abbreviation	mmol/g$_{CDW}$
Acetyl CoA	accoa	0.000279
CoA	coa	0.000168
Succinyl CoA	succoa	0.000098
Malonyl CoA	malcoa	0.000031
NAD	nad	0.001787
NADH	nadh	0.000045
NADP	nadp	0.000112
NADPH	nadph	0.000335
Tetrahydrofolate	thf	0.000223
5,10-Methylenetetrahydrofolate	mlthf	0.000223
5-Methyltetrahydrofolate	5mthf	0.000223
Thiamine diphosphate	thmpp	0.000223
Pyridoxal-5'-phophate	pydx5p	0.000223
Adenosylcobalamin	adocbl	0.000223
Undecaprenyl diphosphate	udcpdp	0.000055
Formyltetrahydrofolate	10fthf	0.000223
Chorismate	chor	0.000223
S-Adenosyl-L-methionine	amet	0.000223
Riboflavin	ribflv	0.000223
Biotin	btn	0.000002
Menaquinone-4	Mqn4	0.000223

Conclusions

Based on the above data, the formation of 1 g of dry cell mass of BB-12 (including growth-associated maintenance energy requirements, GAM) is defined by the following equation:

0.506247 ala-L[c] + 0.294202 arg-L[c] + 0.166414 asn-L[c] + 0.310305 asp-L[c] + 0.048356 cys-L[c] + 0.173330 gln-L[c] + 0.289302 glu-L[c] + 0.370865 gly[c] + 0.116536 his-L[c] + 0.258318 ile-L[c] + 0.419257 leu-L[c] + 0.174719 lys-L[c] + 0.130209 met-L[c] + 0.166992 phe-L[c] + 0.221918 pro-L[c] + 0.273745 ser-L[c] + 0.277573 thr-L[c] + 0.062332 trp-L[c] + 0.128981 tyr-L[c] + 0.374916 val-L[c] + 0.024416 datp[c] + 0.037396 dctp[c] + 0.037396 dgtp[c] + 0.024416 dttp[c] + 0.040848 ctp[c] + 0.052715 gtp[c] + 0.029901 utp[c] + 0.031402 atp[c] + 0.154865 cps_BIF[c] + 0.086043 peptido_BIF[c] + 0.002931 lipota_BIF[c] + 0.030386 pglyc_BIF[c] + 0.047424 clpn_BIF[c] + 0.021788 cpd15_BIF[c] + 0.014942 cpd17_BIF[c] + 0.184745 k[c] + 0.012316 nh4[c] + 0.008211 mg2[c] + 0.004927 ca2[c] + 0.007390 fe2[c] + 0.007390 fe3[c] + 0.003284 cu2[c] + 0.003284 mn2[c] + 0.003284 mobd[c] + 0.003284 zn2[c] + 0.003284 cobalt2[c] + 0.004927 cl[c] + 0.000279 accoa[c] + 0.000168 coa[c] + 0.000098 succoa[c] + 0.000031 malcoa[c] + 0.001787 nad[c] + 0.000045 nadh[c] + 0.000112 nadp[c] + 0.000335 nadph[c] + 0.000223 thf[c] + 0.000223 mlthf[c] + 0.000223 5mthf[c] + 0.000223 thmpp[c] + 0.000223 pydx5p[c] + 0.000223 adocbl[c] + 0.000055 udcpdp[c] + 0.000223 10fthf[c] + 0.000223 chor[c] + 0.000223 amet[c] + 0.000002 btn + 0.000223 ribflv[c] + 20.031402 atp[c] + 15.234885 h2o[c]
→ 20 adp[c] + 20 h[c] + 19.995895 pi[c] + 0.27849 ppi[c] + 1 g biomass

The suffix ‘[c]’ is appended to metabolites abbreviation to indicate their occurrence in the cytoplasm.

The formation of 1 g of dry cell mass of BB-46 (including growth-associated maintenance energy requirements, GAM) is defined by the following equation:

0.507959 ala-L[c] + 0.295197 arg-L[c] + 0.166976 asn-L[c] + 0.311355 asp-L[c] + 0.048519 cys-L[c] + 0.173917 gln-L[c] + 0.290882 glu-L[c] + 0.372120 gly[c] + 0.116930 his-L[c] + 0.259191 ile-L[c] + 0.420675 leu-L[c] + 0.175310 lys-L[c] + 0.130650 met-L[c] + 0.167557 phe-L[c] + 0.222668 pro-L[c] + 0.274671 ser-L[c] + 0.278511 thr-L[c] + 0.062542 trp-L[c] + 0.129147 tyr-L[c] + 0.376184 val-L[c] + 0.024416 datp[c] + 0.037396 dctp[c] + 0.0373963 dgtp[c] + 0.024416 dttp[c] + 0.040848 ctp[c] + 0.052715 gtp[c] + 0.029901 utp[c] + 0.154865 cps_BIF[c] + 0.079033 peptido_BL[c] + 0.002931 lipota_BIF[c] + 0.030386 pglyc_BIF[c] + 0.047424 clpn_BIF[c] + 0.021788 cpd15_BIF[c] + 0.014942 cpd17_BIF[c] + 0.175525 k[c] + 0.011702 nh4[c] + 0.007801 mg2[c] + 0.004681 ca2[c] + 0.007021 fe2[c] + 0.007021 fe3[c] + 0.003120 cu2[c] + 0.003120 mn2[c] + 0.003120 mobd[c] + 0.003120 zn2[c] + 0.003120 cobalt2[c] + 0.004681 cl[c] + 0.000279 accoa[c] + 0.000168 coa[c] + 0.000098 succoa[c] + 0.000031 malcoa[c] + 0.001787 nad[c] + 0.000045 nadh[c] + 0.000112 nadp[c] + 0.000335 nadph[c] + 0.000223 thf[c] + 0.000223 mlthf[c] + 0.000223 5mthf[c] + 0.000223 thmpp[c] + 0.000223 pydx5p[c] + 0.000223 adocbl[c] + 0.000055 udcpdp[c] + 0.000223 10fthf[c] + 0.000223 chor[c] + 0.000223 amet[c] + 0.000223 ribflv[c] + 0.000223 mqn4[c] + 0.000002 btn[c] + 20.031402 atp[c] + 15.218768 h2o[c]
→ 20 adp[c] + 20 h[c] + 19.996099 pi[c] + 0.278490 ppi[c] + 1 g biomass

References

1. Meile, L. et al. *Bifidobacterium lactis* sp. nov., a moderately oxygen tolerant species isolated from fermented milk. *Syst. Appl. Microbiol.* **20**, 57–64 (1997).

2. Schleifer, K. H. & Kandler, O. Peptidoglycan types of bacterial cell walls and their taxonomic implications. *Bacteriol. Rev.* **36**, 407–477 (1972).

3. Bezkorovainy, A. & Miller-Catchpole, R. Biochemistry and Physiology of Bifidobacteria (CRC Press, Boca Raton, 1989).

4. Op den Camp, H. J. M., Veerkamp, J. H., Oosterhof, A. & Halbeek, H. Van. Structure of the lipoteichoic acids from *Bifidobacterium bifidum spp. pennsylvanicum*. *Biochem. Biophys. Acta* **795**, 301–313 (1984).

5. Feist, A. M. et al. A genome-scale metabolic reconstruction for *Escherichia coli* K-12 MG1655 that accounts for 1260 ORFs and thermodynamic information. *Mol. Syst. Biol*. **3**, 1–18 (2007).

6. Orth, J. D. et al. A comprehensive genome-scale reconstruction of *Escherichia coli* metabolism-2011. *Mol. Syst. Biol*. **7**, 1–9 (2011).

Supplementary Note 2: Type III cycles in iAZ480 and iMS520

GALU: UTP-glucose-1-phosphate uridylyltransferase	H^+ + Glucose-1-phosphate + UTP <=> UDPglucose + Diphosphate
GALT: Galactose-1-phosphate uridylyltransferase	Galactose-1-phosphate + H^+ + UTP <=> Diphosphate + UDPgalactose
UGLT: UDPglucose--hexose-1-phosphate uridylyltransferase	Galactose-1-phosphate + UDPglucose <=> Glucose-1-phosphate + UDPgalactose
DADK: Deoxyadenylate kinase	ADP + dADP <=> ATP + dAMP
GK1: Guanylate kinase (GMP:ATP)	ATP + GMP <=> ADP + GDP
GK2: Guanylate kinase (GMP:dATP)	dADP + GDP <=> dATP + GMP
ADKd: Adenylate kinase	dAMP + dATP <=> 2 dADP
PGI: Glucose-6-phosphate isomerase	Glucose-6-phosphate <=> Fructose-6-phosphate
G6PI: Glucose-6-phosphate isomerase	Glucose-6-phosphate <=> beta-Glucose-6-phosphate [c]
G6PI3: Glucose-6-phosphate isomerase	beta-Glucose-6-phosphate <=> Fructose-6-phosphate
GLBRAN2: 1,4-alpha-glucan branching enzyme	Glycogen -> Branched Glycogen
GLDBRAN2: Glycogen debranching enzyme	Branched Glycogen -> Glycogen [c]
G3PD1: Glycerol-3-phosphate dehydrogenase	Dihydroxyacetone-P. + H^+ + NADH <=> Glycerol-3-phosphate + NAD
G3PD2: Glycerol-3-phosphate dehydrogenase	Glycerol-3-phosphate + NADP <=> Dihydroxyacetone-P. + H^+ + NADPH
NADTRHD: NAD transhydrogenase	NAD + NADPH -> NADH + NADP
ADK1: Adenylate kinase	amp + ppi <=> adp + ppi

ADK2: Adenylate kinase	amp + atp <=> 2 adp
PPK2: Polyphosphate kinase	atp + ppi -> adp + ppi

Supplementary Note 3: Examples of gap filling

During the manual curation of the metabolic network of BB-46 the reaction of glycerate kinase (GLYCK), which catalyzes the phosphorylation of glycerate to 3-phospho-D-glycerate, was added to the reconstruction based on the annotation of the gene locus I3242_04010. However, glycerate was not produced by any reaction in the original network reconstruction. The gene locus I3242_02515 encodes for an aldehyde dehydrogenase. Aldehyde dehydrogenase has a wide substrate specificity and can produce glycerate when glyceraldehyde serves as a substrate of the reaction (GLYALDDr) (Brenda, 26.07.2018). To fill the gap, the reaction of aldehyde dehydrogenase acting on acetaldehyde was added and assigned to the gene locus I3242_02515 (ALDD2x). The consumed glyceraldehyde is produced by alcohol dehydrogenase from glycerol (ALCD19), which is formed in the reaction catalyzed by cardiolipin synthase (CLPNS2_BIF). In contrast to BB-46, BB-12 does not possess a gene annotated as glycerate kinase and the model needs a glycerol demand reaction to run.

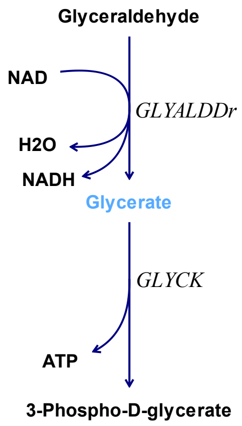

ADPglucose, which is produced in the reaction of glucose-1-phosphate adenylyltransferase (GLGC), was identified as a dead end metabolite in the metabolic networks of BB-12 and BB-46. However, ADPglucose might be converted by glycogen/starch synthase, which is encoded by gene I3242_04075 in BB-46 and BIF_00645 in BB-12 according to genome annotation. The glycogen/starch synthase catalyzes the addition of ADPglucose to a chain of glucose residues, linked by 1,4-alpha glycosidic bonds. Its metabolic function was added as glycogen synthase to the metabolic networks (GLCS1). Synthesized oligosaccharides, such as glycogen, might function as prebiotics for other (bifido-)bacteria in the colon of the host[1]. The genomes of BB-12 and BB-46 include additional gene loci annotated as enzymes that are associated to glycogen metabolism. The function of a glycogen debranching enzyme (GLBRAN2), a glycogen phosphorylase (GLCP2) and a 1,4-alpha-glucan branching enzyme (GLDBRAN2) were added to the GEMs. Based on this set of genes, BB-12 and BB-46 may be able to use glycogen as carbon source, as it was previously shown for *B. longum* subsp. *longum* LMG13197[2].

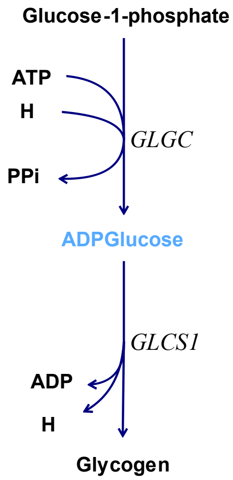

During the process of gap filling, the *Bifidobacterium*-specific biomass objective function was modified. Initially, the reactions of all aminoacyl-tRNA synthetase, catalyzing the attachment of amino acids to their cognate tRNAs, were blocked as the produced aminoacyl-tRNAs were not consumed in the GEMs. However, as these reactions are of metabolic nature, they should be considered for GEMs simulations. Therefore, the free amino acids in the biomass reactions were changed to the respective aminoacyl-tRNAs[3,4].

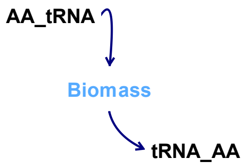

The vitamin biotin was identified as a dead-end metabolite in the draft reconstructions. The reconstructions included an exchange reaction and a transport reaction of biotin into the cell, however, biotin was not consumed by any reaction in the GEM. Biotin is known to be a prosthetic group for enzymes that catalyze carboxylation reactions, such as acetyl carboxylase and bifidobacteria have an absolute requirement for biotin[5]. Therefore, biotin was added to the Bifidobacterium-specific biomass objective function belatedly. The concentration of biotin was adapted from the *i*JO1366 genome-scale metabolic GEM of *E. coli*[6].

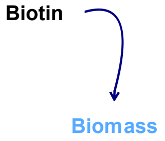

The reaction of adenosylhomocysteinase (AhCi) catalyzing the hydrolysis of S-adenosyl-L-homocysteine to L-homocysteine and adenosine was initially blocked in the metabolic network of BB-46 as the substrate S-adenosyl-L-homocysteine, an intermediate in the biosynthesis of cysteine and adenosine, was not produced in any reaction in the reconstructions. S-adenosyl-L-homocysteine is formed by demethylation of S-adenosyl-L-methionine. For example, S-adenosyl-L-methionine acts as a methyl group donor during the biosynthesis of cyclopropane-19:0 fatty acids, known to be produced by bifidobacteria[7]. Initially, we included the biosynthesis of branched anteiso-C19:0 fatty acids representative for the biosynthesis of cyclopropane-19:0 fatty acids in the reconstructions as anteiso-C19:0 fatty acids have almost the same molecular formula as cyclopropane-19:0 fatty acids ($C_{19:0}$: $C_{19}H_{37}O_2$; cyclo-19:0: $C_{19}H_{35}O_2$). However, the biosynthesis of anteiso-C19:0 includes different enzymes than the biosynthesis of cyclopropane-19:0 fatty acids. The last step of cyclopropane fatty acid biosynthesis is catalyzed by cyclopropane-fatty acid synthase (CFAS_181), which

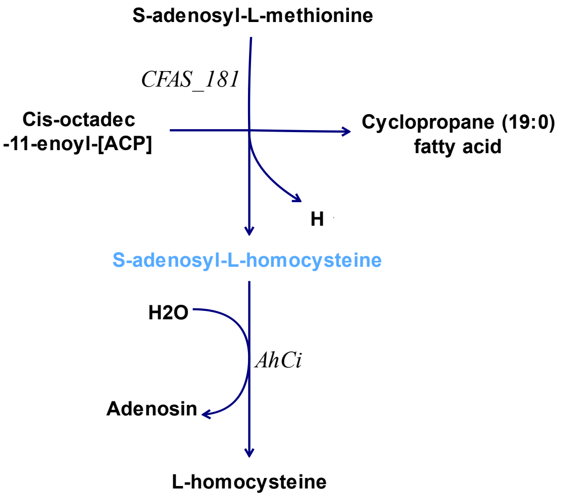

transfers a methyl group from S-adenosyl-methionine to the double bond of an unsaturated fatty acid chain forming a cyclopropane derivate and S-adenosyl-L-homocysteine. Both, the genomes of BB-46 and BB-12 contain a gene which is annotated as cyclopropane fatty acid synthase. During gap-filling, the reaction was added with cis-octadec-11-enoyl-[acyl-carrier protein] (n-C-18:1) as the substrate, resulting in the formation if cyclopropane-19:0. The reaction details were adapted from the genome-scale metabolic GEMs of *Oenococcus oeni* iSM454 and *Leuconostoc mesenteroides* *i*LME620[4,8]. Five reactions from the biosynthesis of the branched anteiso-C19:0 fatty acids were removed from the network reconstructions, including one non-gene-assigned reaction of 2-oxoisovalerate dehydrogenase. The activity of cyclopropane fatty acid synthase in bifidobacteria may be linked to modifications of the fatty acid compositions in the cell membrane, which lowers the fluidity of the membrane and allow the cells to adapt to environmental stressors[9]. Based on genome annotation, BB-12 does not possess a gene for adenosylhomocysteinase. Therefore, the inclusion of cyclopropane fatty acid biosynthesis to the BB-12 model resulted in the need for a demand reaction for S-adenosyl-homocysteine.

The pathway of histidine biosynthesis includes the reaction of histidinol phosphatase (HISTP) as non-gene assigned reaction. This reaction was added to the models as no previous studies showed a requirement for histidine of bifidobacteria.

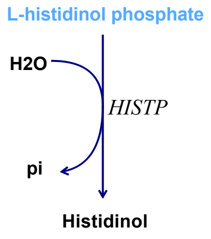

The biosynthesis of methionine is closely linked to the formation of cysteine. Homocysteine can serve as the substrate of methionine synthase, which catalyzes the formation of methionine (METS). According to genome annotation, a gene encoding for methionine synthase is missing in the genome of both strains. However, both genomes include a gene annotated as 5-metyhltetrahydropteroyltriglutamate-homocysteine S-methyltransferase (MHPGLUT), which catalyzes the same reaction as methionine synthase but uses 5-metyhltetrahydrpteroyltri-L-glutamate as a cofactor instead of 5-methyltetrahydrofolate. No reaction for the synthesis of 5-methyltetrahydropteroyltri-L-glutamate was found in biochemical reaction databases. However, it was previously reported for algae that 5,10-methylenetetrahydrofolate

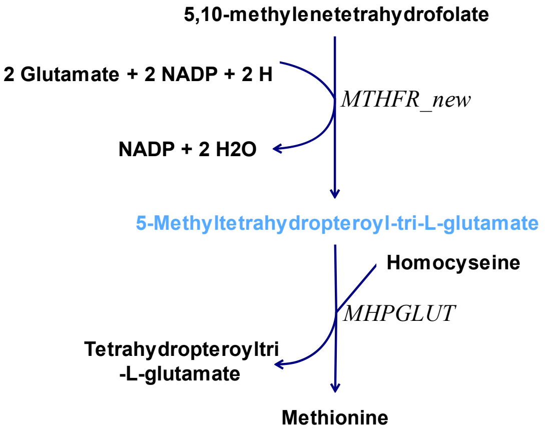

reductase, which are known to convert tetrahydrofolate to 5,10-methylene-tetrahydrofolate (MTHFR2, MTHFR3), can reduce 5,10-methylenetetrafolate directly to 5-methyltetrahydropteroyltri-L-glutamate[10]. Based on this, a new reaction for 5,10-metyhlenentetrahydrofolate reductase (MTHFR_new) was added to the model that produces 5-methyltetrahydropteroyltri-L-glutamate. Furthermore, a demand reaction for the demethylated tetrahydropteroyltri-L-glutamate, which is formed in the reaction of 5-metyhltetrahydropteroyltriglutamate-homocysteine S-methyltransferase, was added and the reaction of methionine synthase was constrained to not carry any flux.

References

1. Pokusaeva, K., Fitzgerald, G. F. & van Sinderen, D. Carbohydrate metabolism in Bifidobacteria. *Genes Nutr*. **6**, 285–306 (2011).

2. Duranti, S. et al. Genomic characterization and transcriptional studies of the starch-utilizing strain *Bifidobacterium adolescentis* 22L. *Appl. Environ. Microbiol*. **80**, 6080–6090 (2014).

3. Teusink, B. et al. Analysis of growth of *Lactobacillus plantarum* WCFS1 on a complex medium using a genome-scale metabolic model. *J. Biol. Chem*. **281**, 40041–40048 (2006).

4. Koduru, L. et al. Genome-scale modeling and transcriptome analysis of *Leuconostoc mesenteroides* unravel the redox governed metabolic states in obligate heterofermentative lactic acid bacteria. *Sci. Rep.* **7**, 15721 (2017).

5. Gyllenberg, H. & Carlberg, G. The nutritional characteristics of the bifid bacteria (*Lactobacillus bifidus*) of infants. Acta Pathol. *Microbiol. Scand*. **44**, 287–292 (1958).

6. Orth, J. D. et al. A comprehensive genome-scale reconstruction of *Escherichia coli* metabolism-2011. *Mol. Syst. Biol*. **7**, 1–9 (2011).

7. Bezkorovainy, A. & Miller-Catchpole, R. *Biochemistry and Physiology of Bifidobacteria* (CRC Press, Boca Raton, 1989).

8. Mendoza, S. N., Cañón, P. M., Contreras, Á., Ribbeck, M. & Agosín, E. Genome-scale reconstruction of the metabolic network in *Oenococcus oeni* to assess wine malolactic fermentation. *Front. Microbiol*. **8**, 534 (2017).

9. Yang, X., Hang, X., Zhang, M., Liu, X. & Yang, H. Relationship between acid tolerance and cell membrane in *Bifidobacterium*, revealed by comparative analysis of acid-resistant derivatives and their parental strains grown in medium with and without Tween 80. *Appl. Microbiol. Biotechnol*. **99**, 5227–5236 (2015).

10. Kettles, N. L., Kopriva, S. & Malin, G. Insights into the regulation of DMSP synthesis in the diatom *Thalassiosira pseudonana* through APR activity, proteomics and gene expression analyses on cells acclimating to changes in salinity, light and nitrogen. *PLoS One* **9**, e94795 (2014).

Supplementary Table 2: Optical density at 600 nm (OD_{600}) reached by BB-46 and BB-12 after 70 h in batch cultivations in the newly formulated chemically defined medium with 12 different carbohydrates (10 g/L). The OD_{600} is given as means of three replicates (n = 3) ± standard deviations. The initial OD_{600} was 0.05. Chemically defined medium without any carbohydrate was used as negative control. STD: standard deviation.

**Carbohydrate**	**OD_{600} [-]**	
	**BB-46**	**BB-12**
**No**	0.07 ± 0.01	0.06 ± 0.01
**Arabinose**	2.83 ± 0.13	0.08 ± 0.16
**Fructose**	4.74 ± 0.15	0.11 ± 0.01
**Galactose**	3.90 ± 0.18	1.1 ± 0.61
**Glucose**	4.61 ± 0.18	4.51 ± 0.04
**Lactose**	4.57 ± 0.13	5.23 ± 0.16
**Maltose**	4.11 ± 0.20	4.42 ± 0.09
**Mannose**	0.07 ± 0.00	0.06 ± 0.01
**Melibiose**	3.61 ± 0.20	3.47 ± 0.39
**Raffinose**	4.05 ± 0.09	5.7 ± 0.05
**Ribose**	0.78 ± 0.16	2.33 ± 0.06
**Sucrose**	4.39 ± 0.42	6.26 ± 0.14
**Xylose**	1.71 ± 0.05	0.1 ± 0.02
